# In vitro assessment and phase I randomized clinical trial of anfibatide a snake venom derived anti-thrombotic agent targeting human platelet GPIbα

**DOI:** 10.1038/s41598-021-91165-8

**Published:** 2021-06-03

**Authors:** Benjamin Xiaoyi Li, Xiangrong Dai, Xiaohong Ruby Xu, Reheman Adili, Miguel Antonio Dias Neves, Xi Lei, Chuanbin Shen, Guangheng Zhu, Yiming Wang, Hui Zhou, Yan Hou, Tiffany Ni, Yfke Pasman, Zhongqiang Yang, Fang Qian, Yanan Zhao, Yongxiang Gao, Jing Liu, Maikun Teng, Alexandra H. Marshall, Eric G. Cerenzia, Mandy Lokyee Li, Heyu Ni

**Affiliations:** 1 Lee’s Pharmaceutical Holdings Limited, 1/F, Building 20E, Phase 3, Hong Kong Science Park, Shatin, N.T. Hong Kong SAR China; 2 Zhaoke Pharmaceutical Co. Limited, Hefei, China; 3 Department of Laboratory Medicine, Keenan Research Centre for Biomedical Science, Li Ka Shing Knowledge Institute, St. Michael’s Hospital, Unity Health Toronto, Toronto, Canada; 4 Toronto Platelet Immunobiology Group, Toronto, Canada; 5 Department of Laboratory Medicine and Pathobiology, University of Toronto, Toronto, Canada; 6 Canadian Blood Services Centre for Innovation, Toronto, Canada; 7 Wannan Medical College First Affiliated Hospital, Yijishan Hospital, Wuhu, China; 8 School of Life Sciences, University of Science and Technology of China, Hefei, China; 9 Department of Physiology, University of Toronto, Toronto, Canada; 10 Department of Medicine, University of Toronto, Toronto, Canada; 11 St. Michael’s Hospital, Room 421, LKSKI-Keenan Research Centre, 209 Victoria Street, Toronto, ON M5B 1W8 Canada

**Keywords:** Biologics, Drug safety, Pharmaceutics, Pharmacology, Target identification, Target validation, Drug discovery, Cardiology, Diseases, Medical research

## Abstract

The interaction of platelet GPIbα with von Willebrand factor (VWF) is essential to initiate platelet adhesion and thrombosis, particularly under high shear stress conditions. However, no drug targeting GPIbα has been developed for clinical practice. Here we characterized anfibatide, a GPIbα antagonist purified from snake (*Deinagkistrodon acutus*) venom, and evaluated its interaction with GPIbα by surface plasmon resonance and in silico modeling. We demonstrated that anfibatide interferds with both VWF and thrombin binding, inhibited ristocetin/botrocetin- and low-dose thrombin-induced human platelet aggregation, and decreased thrombus volume and stability in blood flowing over collagen. In a single-center, randomized, and open-label phase I clinical trial, anfibatide was administered intravenously to 94 healthy volunteers either as a single dose bolus, or a bolus followed by a constant rate infusion of anfibatide for 24 h. Anfibatide inhibited VWF-mediated platelet aggregation without significantly altering bleeding time or coagulation. The inhibitory effects disappeared within 8 h after drug withdrawal. No thrombocytopenia or anti-anfibatide antibodies were detected, and no serious adverse events or allergic reactions were observed during the studies. Therefore, anfibatide was well-tolerated among healthy subjects. Interestingly, anfibatide exhibited pharmacologic effects in vivo at concentrations thousand-fold lower than in vitro, a phenomenon which deserves further investigation.

**Trial registration:** Clinicaltrials.gov NCT01588132.

## Introduction

Platelet adhesion and aggregation at sites of vascular injury are key events in the arrest of bleeding, but also contribute to vascular thrombosis, such as in the atherosclerotic coronary or cerebral arteries, causing heart attack and stroke, the leading causes of morbidity and mortality worldwide^1–4^. The interaction between the platelet receptor glycoprotein (GP) Ibα and von Willebrand factor (VWF) initiates platelet adhesion, particularly under high shear stress^5–7^. Following this initial tethering, platelets become activated allowing integrin αIIbβ3 (GPIIbIIIa) to bind fibrinogen and other ligands, including VWF, mediating platelet aggregation and the formation of a hemostatic plug to stop bleeding or an occluding endovascular thrombus, causing diseases^2,8–10^. Notwithstanding the essential αIIbβ3 contribution to thrombogenesis, the GPIbα-VWF interaction remains critical for pathological endovascular growth of occlusive thrombi at sites of arterial stenosis where blood flows with wall shear rates that may exceed 40,000 s^−1^, corresponding to shear stresses exceeding 1600 Pa^11^.

Currently, aspirin, clopidogrel, and αIIbβ3 antagonists are the primary anti-thrombotic agents used in the long-term treatment of cardiovascular thrombotic disorders^12–15^. Several αIIbβ3 inhibitors, such as abciximab, eptifibatide, and tirofiban, have been in use for over 20 years^15,16^. They are effective anti-platelet and antithrombotic agents, but may induce thrombocytopenia and severe bleeding in some patients^17,18^. To date, no drug targeting GPIbα has been successfully developed, despite favorable arguments supporting such an approach^19–21^, as outlined above, in particular, the likely selective advantage of a GPIbα antagonist in conditions involving arterial thrombosis.

Snaclecs (snake C-type lectins) are a subset of non-enzymatic proteins isolated from venom of different snakes and have been evaluated for their therapeutic potential, including as antithrombotic agents for decades; however, none have yet successfully entered clinical practice^22,23^. Recently, studies have focused on agkisacucetin (generic name: anfibatide)^21,24^ and agkisacutacin^25–27^, two GPIb-binding snaclecs purified from the venom of *Deinagkistrodon acutus* (*D. acutus*, also known as *Agkistrodon acutus*) with different sequences listed in the UniProt database (Anfibatide α-subunit: Q8JIV9, and β-subunit: Q8AYA3; agkisacutacin α-subunit: Q9IAM1, and β-subunit: Q8JIW1). Anfibatide and agkisacutacin, like other snaclecs, consist of α- and β-subunits that are disulfide-linked, forming αβ heterodimers. Interestingly, anfibatide contains an unpaired Cys residue, as seen in the crystal structure^24^, which is unusual for snaclecs and may contribute to its unique function through disulfide-interactions with other snaclec subunits or targeted proteins^28^. Anfibatide also contains no Ca^2+^ binding loops^24^, consistent with the lack of interaction with the Vitamin K-dependent carboxylation/gamma-carboxyglutamic (GLA) domains of coagulation factors.

Agkisacutacin was initially identified as a fibrinolytic protein with high sequence homology to coagulation factor IX/X-binding protein (BP) derived from *Trimeresurus flavoviridis* venom^29,30^, but with no effect on the activated partial thromboplastin time (aPTT)^25^. One year later, the same group reported a protein identified with the same name (agkisacutacin) that completely inhibited ristocetin and thrombin (but not collagen or ADP)-induced platelet aggregation, and had no fibrinolytic or anticoagulant activity^26^. The discrepancies between the two reports have not yet been adequately explained; interestingly, however, the properties reported in the latter study closely correspond to the results we obtained with anfibatide^21^. Subsequently, agkisacutacin was reported to prolong prothrombin time (PT) and aPTT, and decrease platelet aggregation induced by collagen, ADP, and thrombin while demonstrating both antithrombotic and thrombolytic activities^26^. After further purification, agkisacutacin was later found to bind platelet GPIbα as well as coagulation factors IX and X, and inhibit GPIbα-VWF dependent ristocetin-induced platelet aggregation^27^. Thus, although anfibatide and agkisacutacin share the properties of binding to and inhibiting VWF-mediated functions of GPIbα, only anfibatide shows exclusive selectivity for platelet GPIbα.

At least three additional GPIbα-binding snaclecs have been isolated from *Deinagkistrodon acutus* venom; agkicetin C and akitonin, which inhibit platelet function, and agkaggregin, which induces platelet activation. Flavocetin-A and echicetin, isolated from Habu snake venom, are two snaclecs that also inhibit VWF access to GPIbα; however, they can both support and inhibit platelet aggregation^23,24,29,31^. Akitonin and jararaca GPIb-BP, isolated from Bothrops jararaca, are GPIbα antagonists that decrease platelet aggregation in vitro*,* but their in vivo efficacy and bleeding diathesis have not been reported^32,33^.

We previously evaluated properties of purified anfibatide in vitro and in vivo*,* with mouse models of thrombogenesis^21^. We showed that anfibatide specifically inhibits the GPIbα-VWF interaction as well as platelet functions known to depend on it, including ferric chloride- and laser-induced thrombus formation in mesenteric and cremaster muscle arterioles^21,34^. Such findings suggest that anfibatide has the potential of acting as a GPIbα selective antithrombotic agent in humans, as we briefly reported in 2013 annual meeting of American Society of Hematology^35^. Since the GPIbα-VWF interaction is strictly required for thrombogenesis at pathological levels of shear stress, anfibatide may offer an improved risk/benefit ratio compared to αIIbβ3 and VWF antagonists^36^, particularly in the management and treatment of acute coronary syndromes in which acute occlusion involves pathologically elevated shear stress.

Since then, anfibatide has been shown as a promising candidate that could be beneficial for the treatment of ischemic stroke and has a protective effect on cerebral ischemia/reperfusion injury in animal models^37–40^. Also, when anfibatide is administered at the optimal dosage, route, and interval, it is effective in treating spontaneous and bacterial shigatoxin-induced TTP in murine models. These studies may provide the basis for further development of anfibatide for the treatment of acute TTP in humans^41^. In addition, the first balanced expression strain and pilot-scale production of recombinant anfibatide in *P. pastoris* has been reported^42,43^, aiming to solve the quality control difficulties of purifying anfibatide from raw snake venom and the limited supply of this natural resource. Further improvements of recombinant anfibatide are still under development.

This present study evaluates the antithrombotic efficacy and safety of anfibatide in vitro*, *ex vivo with human blood, and after injection and infusion in healthy human subjects—making anfibatide the first GPIbα antagonist tested in humans. Our data suggest that anfibatide may be a potentially safe and effective agent for antithrombotic therapy targeting platelet GPIbα.

## Results

### Purification of anfibatide from *Deinagkistrodon acutus* venom

**Figure 1:**
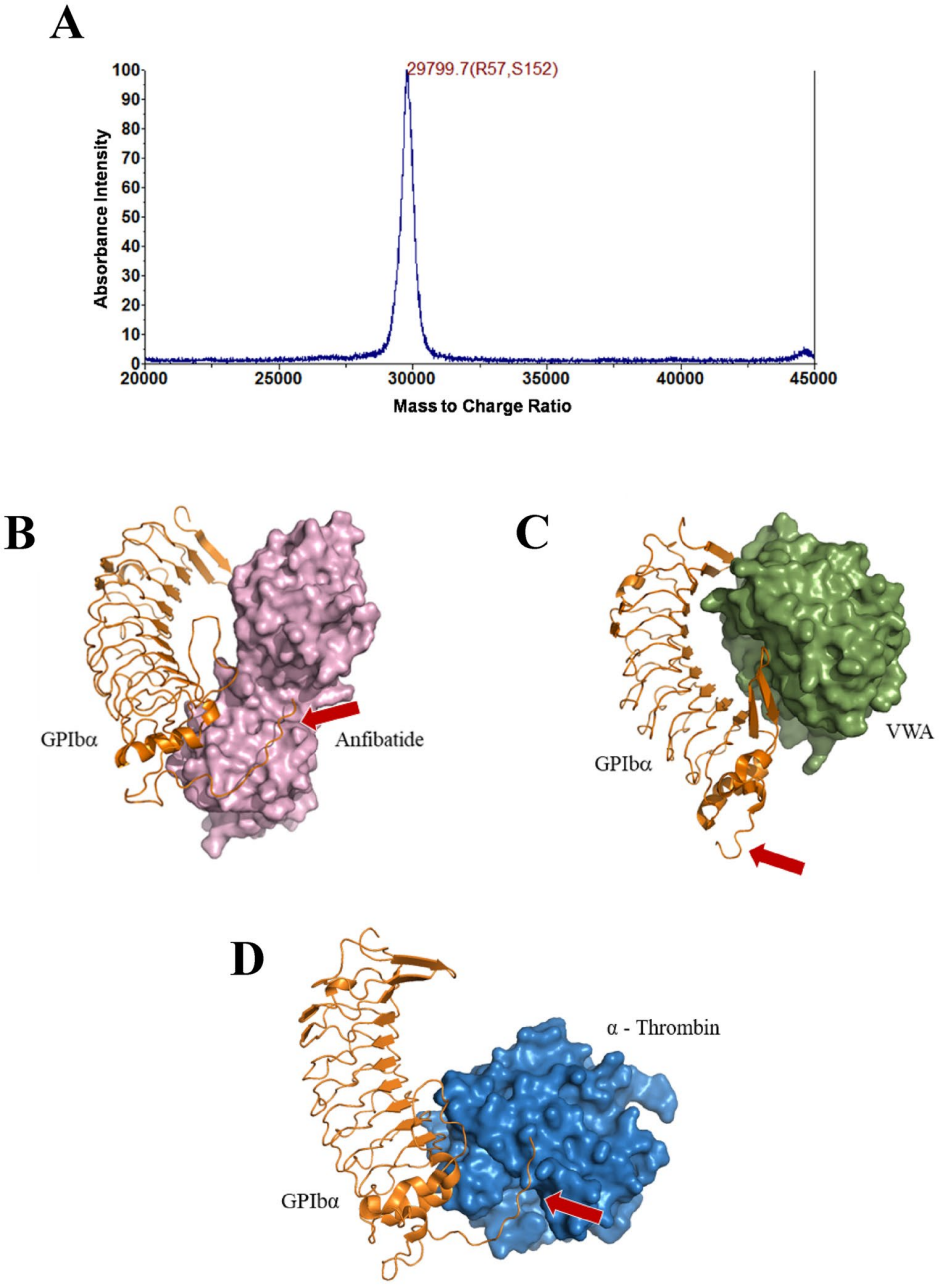
Purification and structure of anfibatide with GPIbα. (**A**) MALDI-TOF mass spectrometry showed a mass to charge ratio (m/z) of 29,799.7. Three-dimensional models of (**B**) structure of anfibatide (purple) integrated with GPIb (orange) and (**C**) VWF-A1 domain (green) and GPIbα complex from PDB entry 1SQ0^45^ (https://www.rcsb.org/structure/1SQ0). (**D**) α-Thrombin (blue) and GPIbα complex from PDB entry 1OOK^44^ (https://www.rcsb.org/structure/1OOK). Red arrows point to the sulfotyrosine region of GPIbα, the α-thrombin binding site. Protein-complex figures generated using Schrodinger PyMol 2 software (https://pymol.org/2/).

Anfibatide was purified from the snake venom (produced by Huangshan Huizhou Research Institute of Snake Venom, Huangshan, China) by anion-exchange chromatography and cation-exchange chromatography followed by immuno-affinity chromatography with a specific anti-anfibatide monoclonal antibody 1B9 (produced by Zhaoke Pharmaceutical Co. Ltd., Hefei, China)^43^. The eluted product was further purified by size exclusion chromatography using a Sephacryl S-100 column. After the four-step purification (Supplementary Methods), MALDI-TOF mass spectrometry analysis showed a single peak, indicative of high purity, with a mass to charge ratio (m/z) of 29,799.7 (Fig. 1A), which corresponds to the theoretical molecular weight of anfibatide of approximately 30 kDa. This single-peak protein was used for subsequent in vitro and ex vivo experiments as well as for the phase I clinical trial.

### In silico modeling of the anfibatide–GPIbα interaction

We have reported the crystal structure of anfibatide^24^. To elucidate the potential binding site of anfibatide on GPIbα, a three-dimensional structural model of the GPIbα-anfibatide complex was generated by in silico molecular docking based on information available in the Protein Data Bank (PDB), 1OOK^44^ for GPIbα, 3UBU^24^ for anfibatide, and superposed to the GPIbα-VWF-A1 domain (VWA) complex (PDB entry 1SQ0^45^). These structural models show close proximity between the anfibatide and VWF-A1 binding sites on GPIbα, implying that anfibatide can act as a competitive inhibitor of the VWF-GPIbα interaction (Fig. 1B,C). Interestingly, in contrast to VWF-A1, this model suggests that anfibatide also interacts with the sulfotyrosine region of GPIbα, the α-thrombin binding site (Fig. 1D).

### Anfibatide binds to GPIbα, inhibiting VWF and thrombin binding

**Figure 2:**
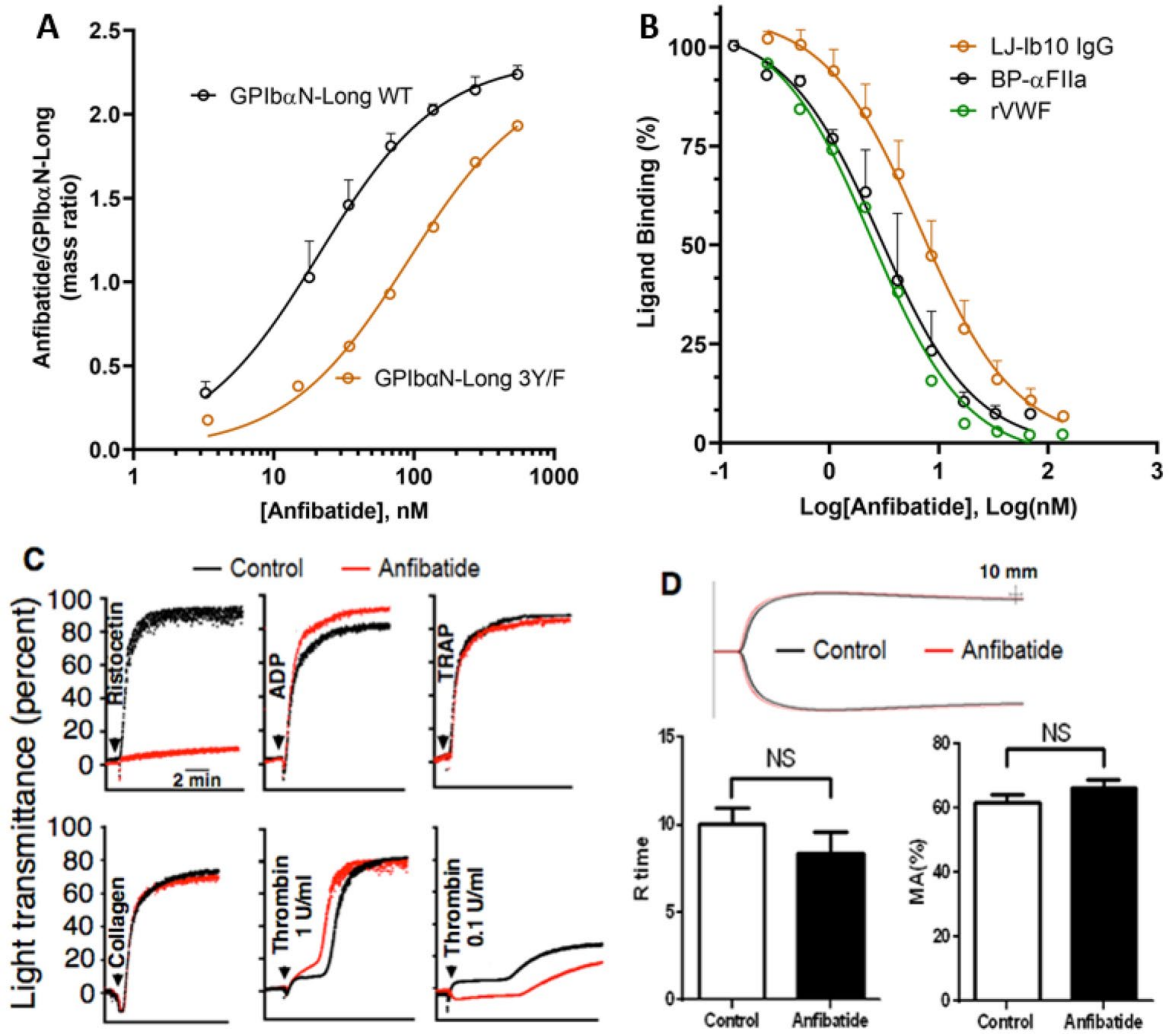
Anfibatide binding to GPIbα prevented VWF and thrombin binding as well as platelet aggregation induced by ristocetin/VWF and low-dose thrombin, but had no effect on blood clotting. (**A**) SPR analysis of anfibatide binding to disulfide-linked dimeric GPIbαN-Long (a chimera of human GPIbα residues − 2 to 288 with 133 residues of SV40 large T antigen) either with wild-type (WT) GPIbα sequence or with Tyr to Phe substitution of residues 276, 278 and 279 (3Y/F). GPIbαN-Long was captured onto the SPR chip by an immobilized anti-SV40-T mAb, followed by anfibatide at increasing concentrations. Data are presented as the ratio of anfibatide/GPIbαN-Long mass bound to the SPR chip during the equilibrium phase (prior to dissociation) and are fit to a one-site ligand binding model. Mass ratio ± SD, N = 3, Black: GPIbαN-Long WT; Orange: GPIbαN-Long 3Y/F. (**B**) Inhibition of α-thrombin (BP-αFIIa), VWF A1 domain or mAb LJ-Ib10 binding to GPIbα on human washed platelets by increasing anfibatide concentrations. Data are presented as % binding, relative to the binding of the ligand at a concentration equal to the K_D_ of the ligand in the absence of anfibatide. The data was fit to the Cheng–Prusoff transformation^79^. Ligand binding % ± SD, N = 3, Black: BP-αFIIa; Orange: LJ-Ib10; Green: rVWF. (**C**) Platelet aggregation was induced by ristocetin (1.2 mg/mL) (P < 0.001), ADP (20 μM) (P > 0.05), TRAP (500 μM) (P > 0.05), or collagen (10 µg/mL) (P > 0.05) in anfibatide-treated (6 µg/mL) PRP or by thrombin (0.1–1 U/mL) in gel-filtered platelets. Curves are from representative light transmission aggregometry plots. (**D**) Clot formation was measured by thromboelastography in anfibatide-treated whole blood. Anfibatide (6 µg/mL) did not significantly alter the time to initial clot formation (R time, min ± SEM) or maximum clot strength (MA ± SEM). NS, no significant difference. Red: anfibatide-treated plasma. Black: untreated plasma (N = 6).

Surface plasmon resonance (SPR) was used to evaluate anfibatide binding to a recombinant chimeric protein formed by residues − 2 to 288 of human GPIbα, followed by 133 residues of the SV40 large T antigen; this disulfide-linked dimeric molecule was designated GPIbαN-Long^46^. Two species of GPIbαN-Long were prepared; one with wild-type (WT) sequence, and one with the mutation of three Tyr residues (276, 278, 279) with Phe (designated 3Y/F), preventing post translational sulfation that normally occurs at these positions and is responsible for contacting thrombin exosite II and exosite I residues^46^. Anfibatide bound to GPIbαN-Long WT with a K_D_ = 21.0 ± 0.9 nM and bound to the 3Y/F mutant GPIbαN-Long with approximately fourfold lower affinity, K_D_ = 90.5 ± 9.2 nM (Fig. 2A). In agreement with the SPR results and structural models, anfibatide inhibited the binding of both recombinant VWF and α-thrombin to GPIbα on the platelet surface producing similar inhibition constants, K_i_ = 1.2 ± 0.5 nM and 1.4 ± 0.1 nM, respectively (Fig. 2B). Moreover, anfibatide inhibited the binding of a monoclonal antibody (mAb) LJ-Ib10 (K_i_ = 3.4 ± 1.1 nM ) that recognizes the human GPIbα sulfotyrosine region^47^ and itself is an inhibitor of thrombin binding (Fig. 2B).

### Anfibatide inhibits platelet aggregation induced by ristocetin and low-dose thrombin, without influencing blood coagulation

Concordant with the results of binding studies, anfibatide specifically blocked VWF-mediated ristocetin-induced platelet aggregation in human platelet-rich plasma (PRP) without interfering with aggregation induced by various doses of ADP, thrombin-receptor activating peptide (TRAP/PAR1AP), collagen, or high dose thrombin (1 U/mL); however, it delayed and decreased aggregation induced by low dose thrombin (0.1 U/mL) (Fig. 2C, Supplementary Figure 2), which confirmed SPR result of Fig. 2B, indicating anfibatide blocked platelet aggregation induced by interaction between GPIbα and low dose thrombin. We also found that anfibatide dose dependently inhibited human platelet aggregation induced by botrocetin (Supplementary Figure 3). Of note, evaluation of whole blood clot formation by thromboelastography (TEG) showed that anfibatide did not significantly influence the time to initial fibrin clot formation (R time) or the mechanical strength of clots (maximum amplitude, MA; Fig. 2D), suggesting the thrombin-GPIbα pathway in blood coagulation is not significantly affected by anfibatide^44,48,49^.

### Anfibatide inhibits thrombus formation and dissolves preformed thrombi in blood perfusion chambers in vitro

**Figure 3:**
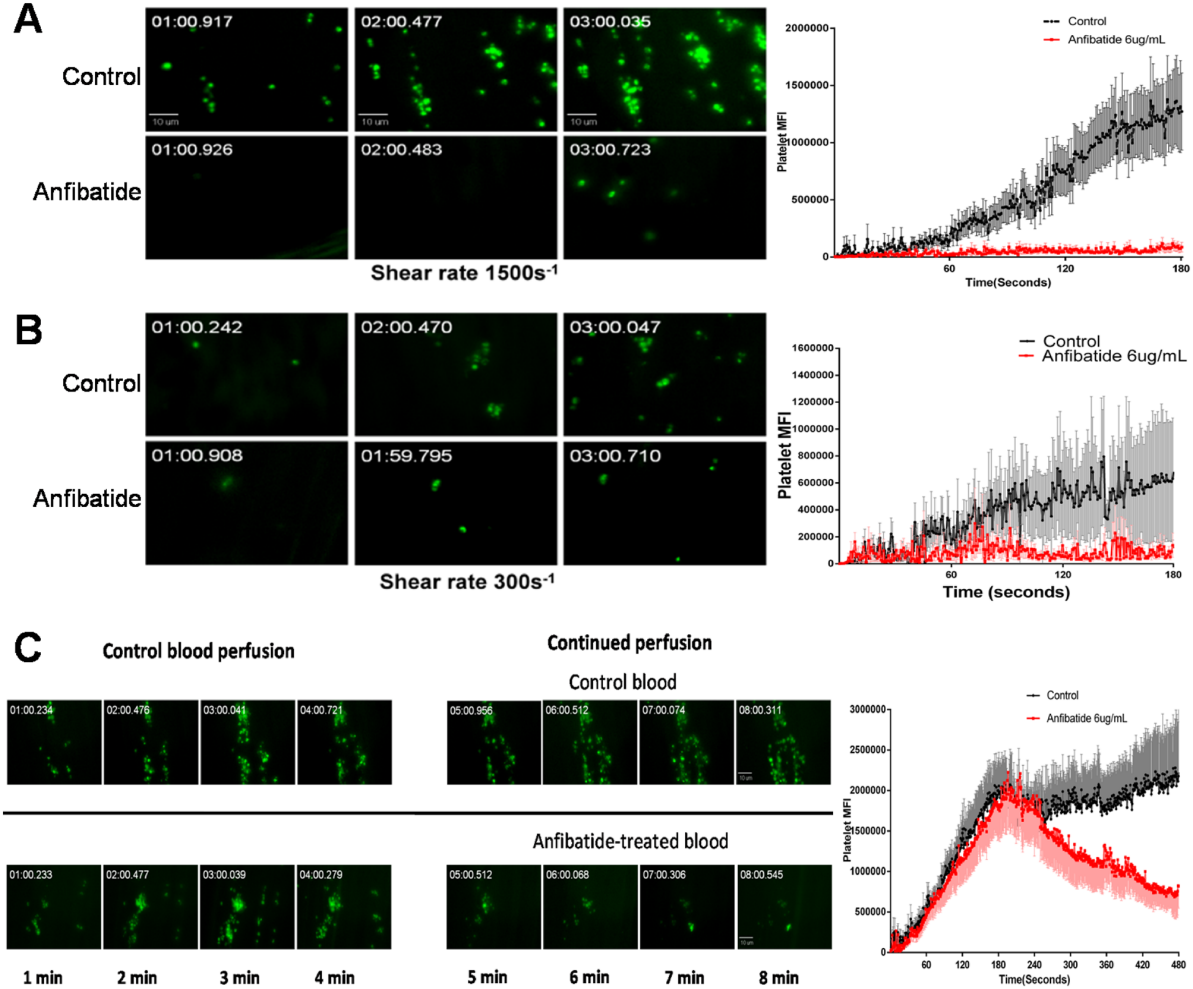
Anfibatide inhibited platelet adhesion, aggregation and thrombus formation and dissolved preformed thrombi under flow conditions. Platelets in heparin-anticoagulated whole blood from healthy volunteers were fluorescently labeled with DiOC6 before perfusion over collagen at the wall shear rate of 1500 s^−1^ (**A**) or 300 s^−1^ (**B**) with or without anfibatide (6 µg/mL). (**C**) Control blood was first perfused at 1500 s^−1^ for 4 min to form thrombi; then, perfusion was continued with control or anfibatide-treated (6 µg/mL) blood. Representative images of fluorescent platelets (Left) are shown along with plots of the platelet mean (± SEM) fluorescence intensity as a function of time (Right; N = 12). P < 0.01 between control and treatment groups in all three figures. Two-tailed Student’s t-test was used to test for significant differences between 2 groups.

The effect of anfibatide on platelet adhesion, aggregation, thrombus formation, and thrombolysis at different shear rates was assessed using an in vitro thrombosis model^9,50–53^ in perfusion chambers coated with collagen, on which soluble VWF from blood quickly anchors. Whole blood from healthy volunteers was perfused over the collagen-coated surfaces with or without the addition of anfibatide, and imaged in real-time by fluorescence microscopy. Anfibatide markedly inhibited platelet adhesion, aggregation, and thrombus formation under high shear rate conditions (1500 s^−1^; Fig. 3A) as well as, albeit less efficiently, at lower shear rate conditions (300 s^−1^; Fig. 3B). Importantly, anfibatide effectively dissolved preformed thrombi when added to the chamber following 4 min of perfusion (Fig. 3C).

### Phase I clinical trial in healthy volunteers

**Figure 4:**
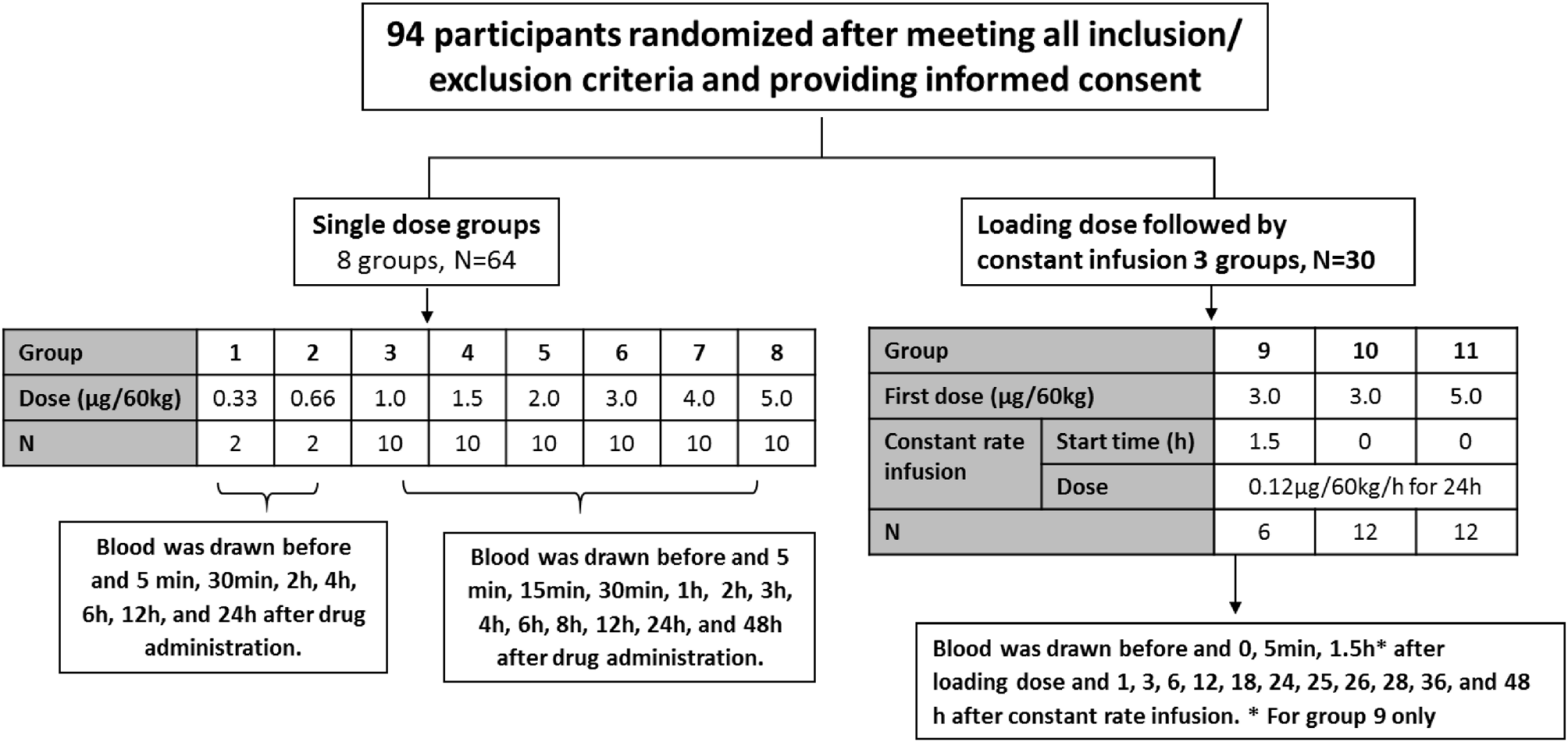
Study design. All randomly-assigned participants were included in the primary outcome analysis. Dose units are μg per 60 kg body weight. N: number of volunteers per group.

This prospective, randomized, open-label phase I clinical trial evaluated the efficacy and safety of anfibatide in healthy human volunteers. In total, 94 healthy male and female volunteers aged 18–28 years were enrolled. Baseline characteristics are summarized in Supplementary Tables 1 to 2. Anfibatide was administered intravenously (*i.v.*) to groups of healthy volunteers either as a single dose bolus (single dose groups 1–8: 0.33, 0.66, 1, 1.5, 2, 3, 4, and 5 µg/60 kg body weight, respectively; *N* = 2–10; Fig. 4) or a bolus (e.g. 3 and 5 µg/60 kg body weight) followed by a constant rate infusion (CRI) at 0.12 μg/60 kg/h for 24 h (multiple dose group 9–11; *N* = 6–12; Fig. 4).

**Figure 5:**
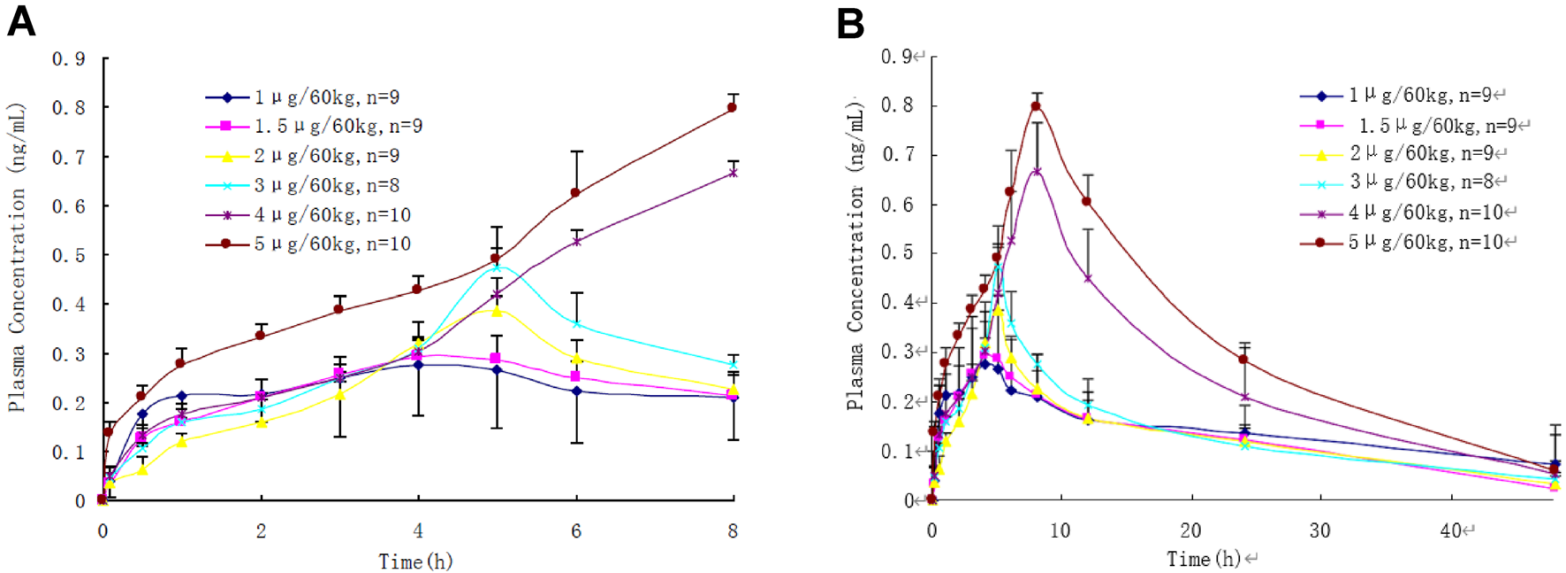
Plasma concentration–time curves of anfibatide in healthy volunteers after single intravenous bolus injection at dose levels of 1, 1.5, 2, 3, 4 and 5 µg/60 kg, respectively (Time = 0–8 h, **A**; Time = 0–48 h, **B**). Mean ± SEM.

Pharmacokinetic parameters were collected as a function of time elapsed from anfibatide administration and dose infused (Fig. 5, Supplementary Tables 3 to 5). There was minimal variation in the blood plasma concentrations of anfibatide among individuals given the same dose. Area under the plasma concentration–time curve increased linearly with increasing doses at ranges between 3 and 5 μg/60 kg (R^2^ = 0.99) (Supplementary Figure 1A,B). Anfibatide elimination was rapid, and the drug was undetectable in plasma samples approximately 48 h after withdrawal (Fig. 5).

**Figure 6:**
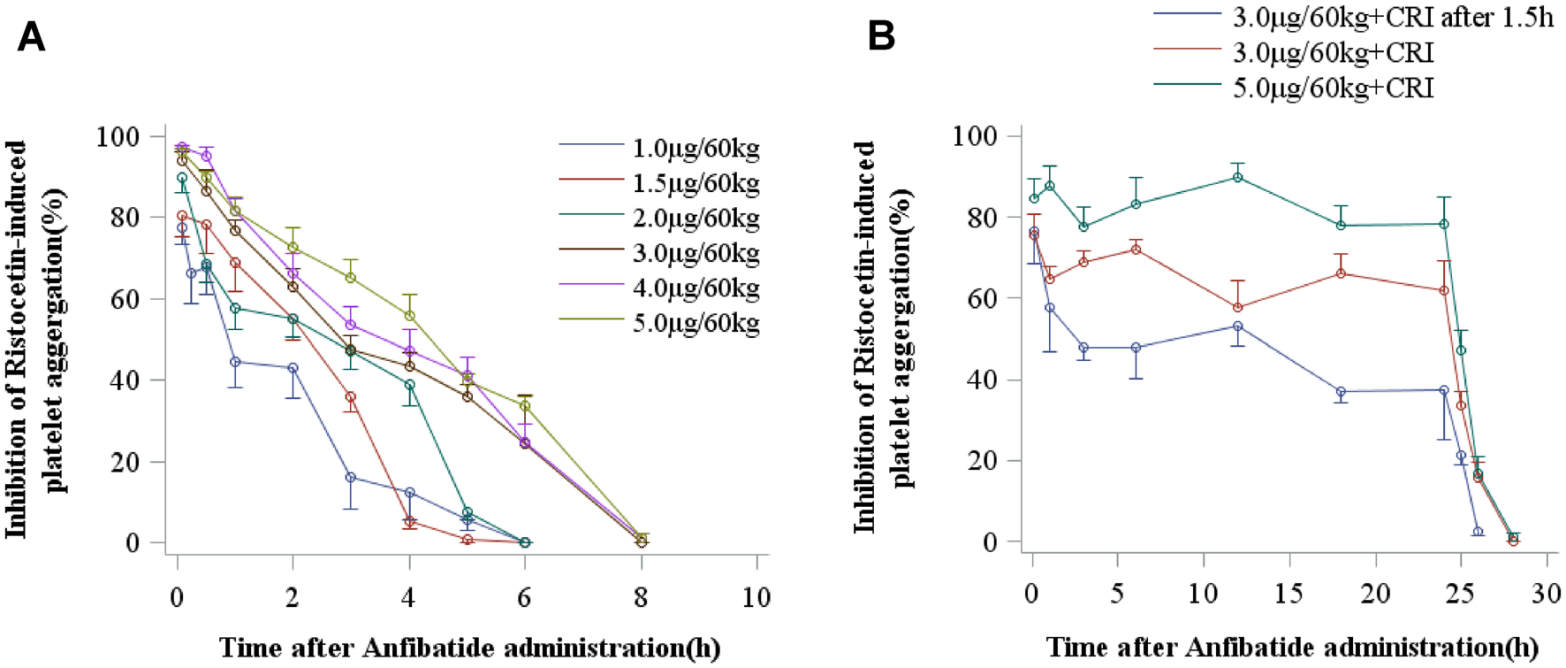
Anfibatide inhibited ristocetin-induced platelet aggregation. Human platelet aggregation induced by ristocetin was studied by platelet aggregometry in single (**A**, N = 8–9/each) and multiple dose groups (**B**, N = 6–12/each). The mean inhibitory rate of anfibatide on platelet aggregation over time has been shown. CRI, constant rate infusion; Mean ± SEM.

To assess the anti-platelet effect of anfibatide at the doses and modes of administration tested in this trial, whole blood samples were collected from participants before and after anfibatide treatment, and platelet aggregation in human PRP was induced ex vivo by ristocetin. Aggregometry results revealed a time- and dose-dependent inhibition of VWF-mediated ristocetin-induced platelet aggregation in blood collected from anfibatide-treated volunteers (Fig. 6A). The anti-platelet effect started immediately after infusion of anfibatide (Fig. 6A,B, Supplementary Tables 6 to 9). Anfibatide disassociation was also fast, and the anti-platelet effect abated within 6–8 h after drug cessation in the single dose groups (Fig. 6A, Table 1). Interestingly, the maximum inhibition occurred immediately after infusion when the plasma concentration of unbound anfibatide was almost undetectable, suggesting most of anfibatide may quickly bind to GPIbα and block the GPIbα-VWF interaction. The plasma anfibatide concentration reached a maximum 6–8 h after anfibatide injection, putatively suggesting that an inactive anfibatide metabolite may gradually be formed and released from platelets, or anfibatide-GPIbα complex shedding from platelet surface, although these hypotheses are required to be further investigated. A constant rate infusion of anfibatide maintained its inhibition (Fig. 6B) and the anti-platelet aggregation effect disappeared within 4 h after infusion (Fig. 6B). Thus, the inhibitory effect of anfibatide on ristocetin-induced platelet aggregation was dose-dependent and reversible.

**Table 1:** Anfibatide pharmacodynamic effects in healthy volunteers.

Groups	Number/group	Emax (%) Mean ± SD	Tmax (h) Mean ± SD	Tmin (h) Mean ± SD	AUEC (%) Mean ± SD
**Single dose groups**					
1 (µg/60 kg)	10	79.5 ± 14.2	0.201 ± 0.171	4.4 ± 1.4	149.4 ± 82.9
1.5 (µg/60 kg)	10	80.7 ± 16.9	0.176 ± 0.289	4.6 ± 0.8	183.4 ± 66.2
2 (µg/60 kg)	10	89.9 ± 11.9	0.085 ± 0.000	5.9 ± 0.3	231.5 ± 72.5
3 (µg/60 kg)	9	92.3 ± 9.1	0.131 ± 0.138	7.1 ± 1.1	299.8 ± 70.9
4 (µg/60 kg)	10	97.8 ± 1.9	0.210 ± 0.200	8.0 ± 0.0	338.9 ± 95.6
5 (µg/60 kg)	10	96.7 ± 2.4	0.210 ± 0.200	8.0 ± 0.0	324.0 ± 90.8
**Multiple dose groups**					
3 µg/60 kg + CRI at 1.5 h	6	81.3 ± 18.4	1.72 ± 2.99	27.5 ± 0.0	1248.7 ± 236.9
3 µg/60 kg + CRI	12	82.2 ± 7.1	8.03 ± 8.58	28 ± 0.0	1694.4 ± 214.7
5 µg/60 kg + CRI	12	94.9 ± 6.7	6.69 ± 8.88	28 ± 0.0	2190.1 ± 303.3

CRI, constant rate infusion; Emax, maximal effect on inhibition of ristocetin-induced platelet aggregation; Tmax, time to Emax; Tmin, time of minimal inhibitory effect on platelet aggregation; AUEC, area under the effect curve.

**Figure 7:**
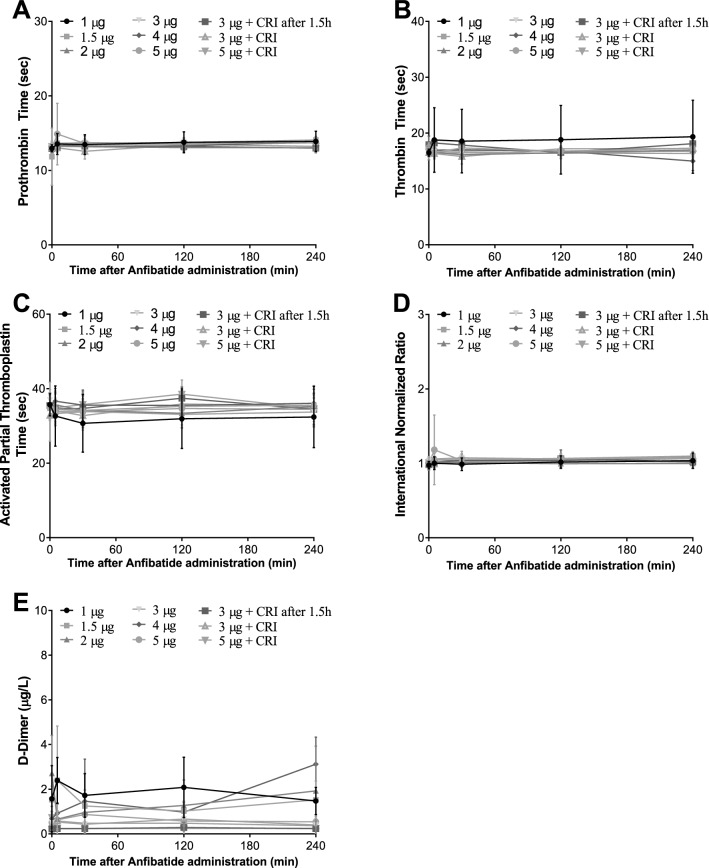
Anfibatide had no significant effect on coagulation measured by prothrombin time (**A**), thrombin time (**B**), activated thromboplastin time (**C**), and international normalized ratio (**D**). anfibatide did not significantly change circulating d-dimers (**E**), representing fibrinolysis. (CRI indicates constant rate infusion). Mean ± SD.

**Figure 8:**
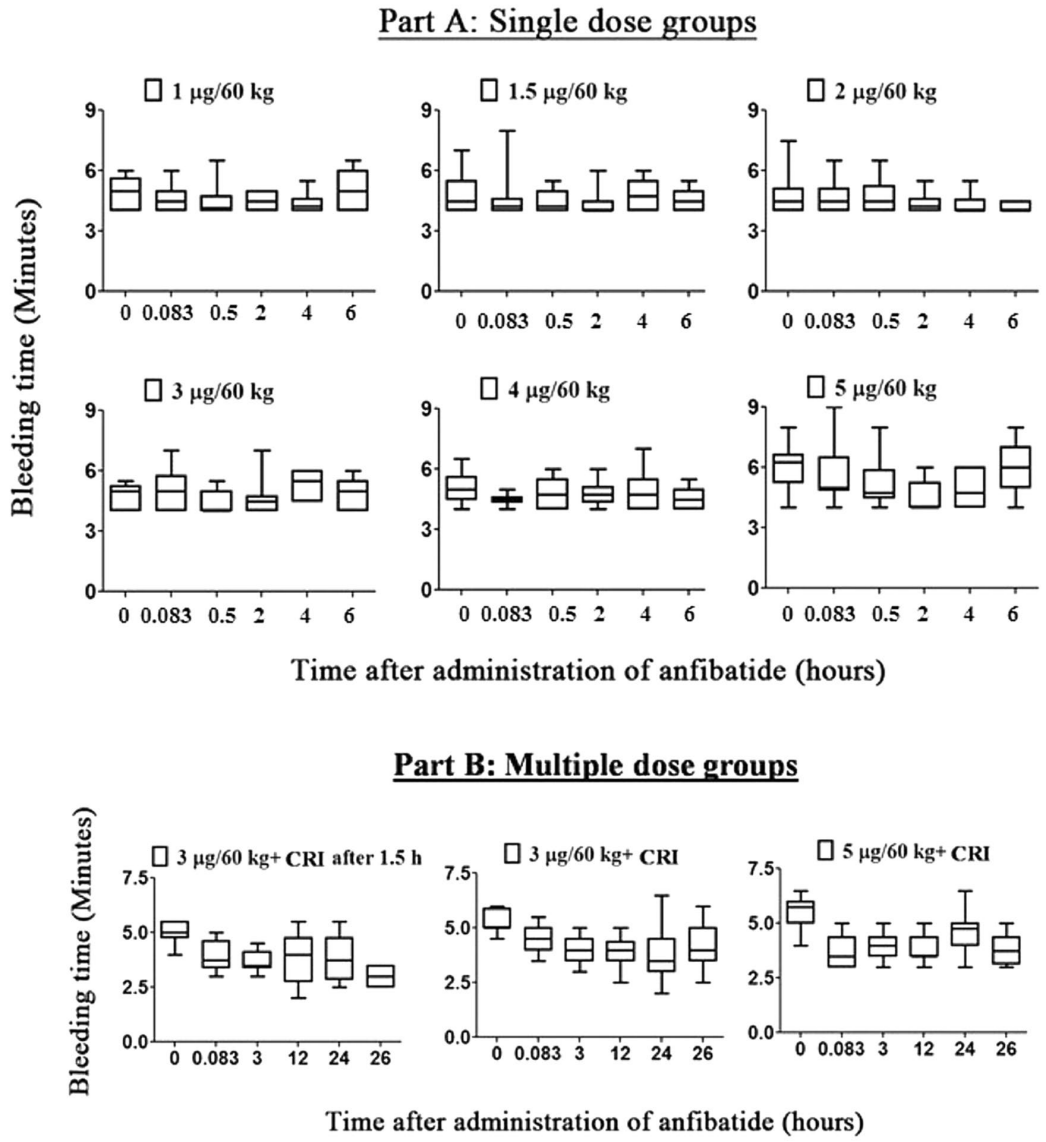
Anfibatide did not significantly prolong bleeding time. Bleeding time was monitored in both single (**A**, N = 8–9/each) and multiple dose groups (**B**, N = 6–12/each), and each bleeding time measured was determined to the nearest 30 s. Majority of the subjects in the single dose groups (except 5 subjects) had a lower bound close to 4 min, while those in the multiple dose groups had a lower bound ranging from 2 to 4 min. None of the subjects in the single and multiple dose groups had a bleeding time of more than 9 min. Bleeding time of all subjects was within the normal range of 2 to 9 min. CRI: constant rate infusion.

Anfibatide did not cause significant platelet count reduction in all participants (Table 2). Furthermore, anfibatide had no noticeable effect on coagulation as measured by thrombin time, prothrombin time, activated thromboplastin time, and international normalized ratio (Fig. 7A–D). Anfibatide did not affect fibrinolysis, as no significant change in circulating D-dimers was detected (Fig. 7E). Importantly, at the doses studied in this trial, anfibatide did not significantly prolong the bleeding time (Fig. 8A,B) and no bleeding symptoms were observed.

**Table 2:** Anfibatide did not significantly change platelet count.

Group no	Dose (µg/60 kg)	Platelet count (× 10^9^/L) (mean ± SD)	
		Before administration of anfibatide	24 h after administration of anfibatide
1	0.33	201.50 ± 12.02	207.50 ± 3.54
2	0.66	226.00 ± 79.20	238.00 ± 94.75
3	1.0	280.80 ± 79.29	269.40 ± 68.05
4	1.5	220.20 ± 69.59	218.90 ± 69.01
5	2.0	240.10 ± 49.59	256.00 ± 48.05
6	3.0	223.00 ± 59.57	224.22 ± 48.62
7	4.0	218.30 ± 47.73	229.30 ± 40.82
8	5.0	219.90 ± 41.92	216.30 ± 41.49
9	3 + CRI at 1.5 h	238.33 ± 58.91	253.00 ± 45.16
10	3 + CRI	232.83 ± 49.12	243.08 ± 47.31
11	5 + CRI	195.33 ± 40.76	213.75 ± 59.31

Platelet counts of each individual were measured before and 24 h after administration of anfibatide in single (Group 1–8, N = 8–9) and multiple dose groups (Group 9–11, N = 6–12).

CRI, constant rate infusion; Mean ± SD.

A thorough safety assessment was performed to determine whether anfibatide caused any systemic adverse events, allergy, or antibody production. There were no statistically significant differences for any vital sign parameters (i.e. body temperature, heart rate, respiratory rate and blood pressure) (Supplementary Tables 10, 11) across all treatment groups. No serious adverse events or allergic reactions were observed during the studies. There were no clinically significant abnormalities of biochemistry and hematology parameters and electrocardiogram during the entire duration of the trial (Supplementary Tables 12 to 18). No anti-anfibatide antibodies were detected in blood samples before or 1 month after anfibatide administration in all treatment groups.

## Discussion

In this study, we demonstrated that anfibatide, a GPIbα-binding snaclec, may represent a novel antithrombotic treatment that does not significantly affect hemostasis under the conditions and doses studied. Through binding to the N-terminus of GPIbα, anfibatide competitively blocked VWF-A1 domain and thrombin binding, and GPIbα-VWF-mediated and low dose thrombin-induced platelet aggregation in vitro. Anfibatide also inhibited ex vivo platelet adhesion and aggregation, and dissolved preformed thrombi when added to the blood before or during perfusion chamber experiments, especially at high shear. The phase I clinical trial demonstrated a dose-related, highly specific pharmacodynamic action of anfibatide. Ristocetin-induced platelet aggregation was inhibited dose-dependently immediately after intravenous infusion. Anfibatide was well-tolerated, as no serious adverse events occurred, and no anti-anfibatide antibodies were detected in these participants. The results of this phase I clinical trial have demonstrated the safety and efficacy of the first promising anti-platelet GPIbα treatment, anfibatide.

The GPIb-IX-V complex plays a pivotal role in initiating and propagating both hemostasis and thrombosis^2,5,22,54,55^. Once activated by pathological levels of shear stress, VWF undergoes conformational changes and binds to the GPIbα receptor^2,56^, which mediates both early platelet adhesion and late platelet aggregation as shear stress escalates before vessel occlusion^7^. GPIbα-VWF engagement can also deliver signals to platelets and activate αIIbβ3, which can synergistically enhance thrombus growth^57^. Therefore, VWF and the GPIbα-IX-V complex have long been considered potential targets for anti-platelet drug development. However, no anti-GPIbα drugs have been developed for clinical practice thus far.

Based on recent evidence from murine model and structural studies^21,24^ we suspect that GPIbα may be a safer and more effective target than VWF. Initial platelet adhesion and thrombus formation were impaired but not entirely prevented in VWF knockout mice^7^. Interestingly and importantly, approximately 50% of the vessels in VWF knockout mice were not completely occluded, which suggests that GPIbα-VWF interaction may be required for late stage platelet aggregation under extremely high shear stress conditions^7^. Those occluded vessels in VWF knockout mice may have decreased blood shear rates by the end of the long/strenuous intravital microscopy experiments. In humans, VWF antagonists (e.g. ARC1779 and ALX-0081, also caplacizumab, the anti-VWF Nanobody) reduce platelet aggregation but prolong cutaneous bleeding time^58–60^, possibly because VWF is a ligand for both GPIbα and αIIbβ3. In contrast to VWF knockout mice, thrombus formation was completely abolished in GPIbα knockout mice^6^; therefore, GPIbα ligands other than VWF may also play a role in platelet aggregation and thrombus formation. Our recent murine study supported this hypothesis; we observed that anfibatide inhibited thrombus formation even in VWF knockout mice^21^. Furthermore, recent studies showed that GPIbα interacts with thrombin, P-selectin, Mac-1, and Thrombospondin-1^22,44,61^. In this study, we observed that anfibatide competitively inhibited α-thrombin binding and inhibited low dose thrombin-induced platelet aggregation. Thus, anfibatide may have additional benefits for anti-thrombotic therapy beyond inhibition of the GPIbα-VWF interaction.

We demonstrated here that GPIbα antagonism by anfibatide inhibited platelet adhesion, aggregation, and thrombus formation in human blood in vitro, especially at high shear. We observed inhibition of ristocetin-induced (i.e. GPIbα-VWF-mediated) platelet aggregation in human blood when anfibatide was added to blood in vitro or administered to volunteers before blood collection. Anfibatide also inhibited low dose thrombin-induced (αIIbβ3-fibrinogen-mediated) platelet aggregation. Furthermore, anfibatide dissolved preformed thrombi in our ex vivo perfusion chamber thrombosis models. Several mechanisms may contribute to this effect of thrombolysis: first, thrombus growth and dissolution are dynamic processes and occur simultaneously under flow conditions; blocking GPIbα-VWF interaction likely prevents platelet recruitment, thus favoring thrombus dissolution over thrombus growth. Second, GPIbα binding to other ligands (e.g. P-selectin, Mac-1, Thrombospondin-1, and some hemostatic/thrombotic factors) may provide additional bridges between adjacent activated platelets to enhance thrombus stability; breaking these bridges may facilitate thrombus dissolution. Third, VWF-GPIbα and thrombin-GPIbα interactions deliver outside-in signaling via the GPIb-IX-V complex which activates newly recruited platelets in the thrombus and likely maintains their αIIbβ3 integrins in the active conformation required for fibrinogen- and other integrin ligand-mediated platelet aggregation^10,62,63^. Interruption of this GPIbα outside-in signaling may reduce the level of platelet and integrin activation. Thus, the inhibition of GPIbα by anfibatide may induce thrombus dissolution, as well as prevent further thrombus growth and vessel occlusion.

Our phase I clinical trial evaluated the pharmacodynamics, pharmacokinetics, safety, and tolerability of anfibatide. Healthy volunteers were treated with a moderate range of anfibatide doses using two different modes of administration: single dose intravenous injection, or single dose injection followed by a 24 h constant rate infusion. In PRP collected from anfibatide-treated participants (as low as 1 μg/60 kg), ristocetin-induced platelet aggregation was inhibited. This in vivo effect seems to be far more potent than our in vitro assays since the doses used in this clinical trial are several thousands times less. It is currently unknown whether other in vivo factors, as also observed during other antithrombotic agent development^64^, can enhance the inhibitory effect of anfibatide on the ristocetin-induced platelet aggregation in these participants. Notably, a recent study showed that the disulfide bonds in the GPIbα ligand binding domain can be catalyzed by protein disulfide isomerase, which affects GPIbα-ligand interactions^28^. Interestingly, anfibatide can interfere in this process and GPIbα-ligand binding^28^, which may synergistically contribute to the inhibitory effect observed in vivo in the human participants.

Pharmacokinetics of anfibatide detected the free (i.e. platelet unbound) plasma anfibatide, which appeared as dose proportional at high doses administered in this study. In healthy volunteers receiving single dose intravenous injection at 1–3 μg/60 kg, the maximum plasma concentration was observed at 4–5 h post-dose. Following administration, anfibatide likely quickly binds to platelet GPIbα in blood circulation and was disassociated over time from the platelet surface. The peak plasma anfibatide was reached 8 h after the injection of higher doses (4–5 μg/60 kg). In addition, the apparent volume of distribution (Vd) was estimated between 3.6 and 8 L, indicating that anfibatide is mainly distributed to the intravascular fluid that reflects a high degree of blood cell or plasma protein binding. Given that anfibatide is transient in the plasma and undergoes fast disassociation, platelet dysfunction is also reversible. Notably, anfibatide-driven inhibition of ristocetin-induced platelet aggregation had a rapid recovery to baseline levels within 4–8 h after drug discontinuation, while plasma anfibatide concentrations remained at moderate to high levels. Although not fully understood, it is possible that plasma anfibatide, after disassociation from platelets may become inactive (metabolite) or may induce platelet GPIbα ectodomain shedding that likely prevents soluble GPIbα-bound anfibatide from binding to other platelets, a hypothesis that deserves future exploration. The relationship between anfibatide concentration, inhibition of the GPIb-IX-V complex, and platelet function was consistent across all doses and dosing regimens. This study suggests a fast onset, potent, and reversible antithrombotic effect of anfibatide among healthy subjects and provides the basis for assessing the non-ST Segment Elevation Myocardial Infarction (NSTEMI) and ST-segment elevation myocardial infarction (STEMI) patients undergoing percutaneous coronary intervention in the phase Ib-IIa and phase II trials.

As the processes involved in hemostasis and thrombosis are similar, the quest for an antihrombotic agent that does not affect hemostasis has been challenging. Current first-line antithrombotic agents target platelet aggregation by COX-1 inhibition (aspirin), αIIbβ3 antagonists (abciximab, eptifibatide and tirofiban), and P2Y12 antagonists (e.g. clopidogrel and prasugrel)^18^. However, aspirin is an irreversible platelet cyclooxygenase inhibitor, which can cause severe bleeding when patients require emergency surgery^65^. In addition, although αIIbβ3 inhibitors represent effective anti-platelet and antithrombotic agents, they often lead to thrombocytopenia and life-threatening bleeding complications, such as cerebral, alveolar, and gastrointestinal system hemorrhages^66–71^. Similarly, prasugrel demonstrated enhanced platelet inhibition, but prevention of ischemic complications was often achieved with increased bleeding complications^72^. As mentioned previously, novel VWF antagonists (e.g. aptamer ARC1779 and nanobody caplacizumab) reduced platelet aggregation but also have bleeding concerns in clinical trials^59,60^. An optimal therapy for acute vascular syndromes would be targeting platelet aggregation at high shear conditions without impairing hemostasis, such as observed with anfibatide. GPIbα antagonism may, therefore, be a more attractive strategy for antihrombotic therapy.

Anfibatide significantly inhibited GPIbα-mediated platelet aggregation at low concentrations without impairing coagulation or prolonging bleeding time. We propose three possible reasons to explain why anfibatide did not affect hemostasis. First, VWF-GPIbα interactions predominantly exist in the presence of high shear stress found in the arterial circulation; thus, GPIbα antagonist may act specifically at sites of thrombosis, and not systemically. This spatial specificity may help to avoid the common hemorrhagic consequences of conventional anti-platelet agents^12,15,73^. Second, the VWF-GPIbα interaction pathway may be independent of (i.e. not interfere) other pathways leading to clot formation, such as platelet activation via collagen or ADP receptors. Third, anfibatide may have no effect on VWF functions in hemostasis that are independent of GPIbα (such as VWF-β3 integrin interaction)^74^. Importantly, no serious adverse events, premature discontinuations due to adverse events, or deaths occurred during the study. Anfibatide did not significantly affect platelet count, and no anti-anfibatide antibodies were detected in the subjects, suggesting that anfibatide may be safe and well-tolerated in healthy individuals.

In summary, this study is the first demonstration of the safety and efficacy of a GPIbα antagonist in healthy human subjects. Anfibatide is a promising GPIb-IX-V complex antagonist that exhibited strong anti-platelet effects, excellent reversibility, and low bleeding tendency in this clinical trial, representing a new therapeutic agent that may advance the treatment of acute coronary syndrome. This study also demonstrates the promising safety profile of anfibatide and provides the basis for the phase Ia-IIb and phase II clinical trials among NSTEMI and STEMI patients, respectively. Further studies will be required to define the optimal dosing strategy for patients with acute coronary syndrome, who often have a high intracoronary thrombus burden and may require a higher therapeutic dosage of anfibatide in order to achieve a higher anti-platelet effect.

## Methods

### Study design

This work comprises an in vitro pre-clinical study, where the interaction between anfibatide with GPIbα was evaluated by surface plasmon resonance and simulated by computer modeling, and functional properties of anfibatide were tested by platelet binding and aggregation studies (*N* = 6) as well as in perfusion chamber models of platelet thrombus formation (*N* = 12). Human platelets or blood samples were randomly allocated to experimental or control groups. The investigators performing the experiments were blinded for the study duration. The sample size was selected based on our previous publications and publications from other groups. All experimental procedures using human blood samples in vitro and ex vivo were approved by the Research Ethics Board of St. Michael’s Hospital—Unity Health Toronto, Toronto, Canada. All data were included in the analysis.

This study also includes a prospective, single-centered, randomized, open-label, and dose-escalating phase I clinical trial (clinicaltrial.gov identifier No. NCT01588132; date first submitted: April 25, 2012 and date first posted: April 30, 2012)^75^, where the safety and tolerability of anfibatide were assessed in 94 healthy volunteers. The phase I clinical trial was conducted at the Yijishan Hospital (Anhui, China) under Investigational New Drug (IND) approval number of 2005L04666 (date of approval: 30/12/2005), assigned by the State Food and Drug Administration (SFDA), now known as the National Medical Products Administration (NMPA) in China. The clinical protocol and its amendments were approved by the Yijishan Hospital Review Board and the study was performed in compliance with the Declaration of Helsinki on medical research involving human subjects and the Guideline for Good Clinical Practice recommended by the NMPA.

Trial subjects meeting the inclusion and exclusion criteria underwent randomization by statisticians according to the random number coding table generated by the SAS software. Written informed consent was obtained prior to participation for all subjects. 93 participants who completed the phase I study were included in the analysis (1 participant dropped out prior to the trial due to personal reasons). All primary data are in the Supplementary Tables.

### In silico modeling of the GPIbα-anfibatide interaction

Based on our X-ray crystallography data^24^, a three dimensional model of the GPIbα-anfibatide complex was simulated using HADDOCK software^76^. The coordinates used in the docking experiments were from Protein Data Bank (1OOK^44^ for GPIbα, 3UBU^24^ for anfibatide). The figure of the GPIbα–VWF-A1 domain complex structure was created from PDB entry 1SQ0^45^ and the figure of the GPIbα–α-thrombin complex is from PDB entry 1OOK. The figures were created using PyMOL software (Schrödinger, LLC).

### Surface plasmon resonance analysis of the GPIbα-anfibatide interaction

Surface plasmon resonance (SPR) was performed to analyze the interaction between GPIbα and anfibatide as was previously done for GPIbα-VWF A1 domain interaction^77^. Briefly, human recombinant GPIbα amino fragment (residues − 2 to 288, with wild-type or with Tyr to Phe substitution of residues 276, 278 and 279 (3Y/F)) fused to 133 residues of the SV40 large T antigen with terminal Cys residues to mediate dimerization were expressed in Drosophila melanogaster S2 cells and purified from culture supernatant. A monoclonal antibody specific for the SV40 sequence (LJ-3A2) was linked to an SPR chip (HC200M, XanTec Bioanalytics). Using a Biacore 3000 SPR instrument and 135 mM NaCl, 20 mM N-2-hydroxyethylpiperazine-N′-2-ethanesulfonic acid (HEPES-buffered saline, pH 7.4) containing 0.005% Tween 20 as the running buffer, GPIbα-SV40 fusion protein was coupled to the mAb immobilized SPR chip. After GPIbα-SV40 immobilization, anfibatide solutions at varying concentrations prepared in running buffer were injected over the chip (association phase) at 75 μL/min for 3 min followed by running buffer for 20 min (dissociation phase). Binding affinities were determined by plotting the steady state SPR determined mass ratios of anfibatide to GPIbα-SV40 (pre-dissociation), against anfibatide concentration, and fitting the data to a one-site ligand binding model using the Graphpad Prism 8.0 software package (https://www.graphpad.com/scientific-software/prism/).

### Anfibatide competitive binding to platelets

Anfibatide platelet binding experiments were adapted from previous experiments detailing FIIa platelet binding^46^. Briefly, blood from healthy human volunteers was collected in tubes containing acid-citrate-dextrose. The tubes were centrifuged at 600*g* for 12 min to prepare platelet-rich-plasma (PRP). The PRP was then centrifuged at 800*g* for 15 min in the presence of 10 μM PG E1 (Enzo Life sciences) and 0.6 U∕mL Apyrase Grade VII (Sigma Aldrich) to obtain a platelet pellet. The pellet was washed once by resuspension and centrifugation in modified Tyrode’s buffer, pH 6.5 (and finally resuspended in the same buffer but at pH 7.4; the platelet count was adjusted to 2 × 10^7^∕mL. The GPIbα ligands, α-thrombin (Haematologic Technologies Inc.) biotin-PPACK active site blocked, BP-αFIIa, mixed with phycoerythrin-conjugated streptavidin, S-PE, recombinant human VWF A1 domain (residues 445–733 expressed in house) mixed with anti-VWF mAb conjugated with FITC or mAb LJ-Ib10 conjugated with FITC, were mixed with aliquots of the platelet suspension to a final concentration of their respective K_D_ (80 nM for BP-αFIIa^46^, 1.5 nM for VWF^77^ and 10 nM for LJ-Ib10^78^) and incubated at room temperature for 30 min. When indicated anfibatide, at concentrations ranging from 0 to 200 nM, was added to the platelet suspensions 5 min before the corresponding GPIbα ligand. Binding was measured by flow-cytometry, without sample dilution, using a FACS Calibur II equipped with a 488 nm argon laser and the software CellQuest for data evaluation (Becton Dickinson and Company). Results were expressed as geometric mean of the fluorescence intensity (MFI) of 10,000 events and analyzed with GraphPad Prism. Data represented as % binding relative to the MFI of the GPIbα ligands at their K_D_ concentration in the absence of anfibatide. Inhibition constants were determined by the Cheng–Prusoff transformation^79^ using the Graphpad Prism 8.0 software package (https://www.graphpad.com/scientific-software/prism/).

### Platelet aggregometry

Human platelet-rich plasma (PRP) and gel-filtered platelets were prepared from sodium-citrate anti-coagulated whole blood by centrifugation as described^7–9,80,81^. Platelet aggregation in PRP was induced by ristocetin (1.2 mg/mL), adenosine diphosphate (ADP, 10–20 µM), or thrombin receptor activating peptide (TRAP, 500 µM), or collagen (10–20 µg/mL; Nycomed Pharma, Germany) , and in gel-filtered platelets was induced by human thrombin (0.1–1.0 U/mL) with or without anfibatide (6 µg/mL) using a computerized Chrono-log aggregometer (Chrono-Log Corporation, USA), as described^4,7–9,21^.

### Thromboelastography (TEG)

To study hemostatic clot formation and strength, fresh whole blood was tested on a TEG 5000 Analyzer (Hemoscope, Haemonetics Corp., USA) as we previously described^4,82^. Briefly, sodium-citrated control or anfibatide-treated (6 μg/mL) whole blood (340 μL) from each volunteer (N = 6) was mixed with CaCl_2_ (20 μL, 0.2 M) and studied in parallel. Time until initial clot formation (R time) and maximum amplitude (MA), which reflects maximum strength of the platelet–fibrin clot, were calculated. Experiments were halted after clot forming parameters were calculated or after 1.5 h, whichever occurred first.

### In vitro perfusion chamber

To measure platelet adhesion, aggregation, and thrombus formation at different shear rates, whole blood from 12 healthy volunteers was perfused over a type I collagen-coated surface using *an *ex vivo perfusion chamber system, as described^9,50–52^. Briefly, rectangular microcapillary glass tubes (0.1 × 1 mm) were coated with Horm collagen (100 μg/mL, overnight, 4 °C; Nycomed Linz, Austria). Anti-coagulated whole blood (heparin 15 U/mL) from healthy donors was fluorescently-labeled with DiOC6 (1 μM, 10 min, 37 °C; Sigma). Then, control or anfibatide-treated (6 μg/mL) whole blood was perfused over the collagen-coated surface at shear rates of 300 s^−1^ and 1500 s^−1^ for 3 min using a syringe pump (Harvard Apparatus, USA). Platelet accumulation and thrombus formation were recorded in real-time under a Zeiss Axiovert 135-inverted florescent microscope (60 ×/0.90 NA water objective). Quantitative dynamics of platelet fluorescence intensity were acquired using Intelligent Imaging Innovations SlideBook version4.1 software (https://slidebook.software.informer.com/4.1/).

Thrombolysis was studied at high shear flow conditions (1500 s^−1^). Control whole blood was perfused for 4 min to form thrombi on collagen as above. Then, without interruption, perfusion was continued for an additional 4 min with control or anfibatide-treated (6 μg/mL) whole blood. Thrombus formation and subsequent thrombolysis were analyzed as above.

### Phase I clinical trial protocol

Trial subjects were screened for the following inclusion and exclusion criteria.

- Inclusion Criteria were: (1) Healthy volunteers, male and female, aged 18–28 years, (age difference in each group < 10 years); (2) Body mass index (BMI) between 19 and 24 (body weight difference in each group < 10 kg); (3) No history of heart, liver, kidney, digestive tract, nervous system and metabolic disorder, or ulcer, significant hemorrhage, without the history of drug allergy and postural hypotension; (4) No abnormalities in medical examinations; (5) Have not taken any medications within 2 weeks before the study; (6) Willing to participate in the study and give a signed informed consent form^75^.
- Exclusion Criteria were: (1) History of HBV or HCV infection; (2) Addicted to smoking or alcohol; (3) Women during pregnancy, lactation or menstrual period; (4) Past history of hemoptysis, bloody stool, bleeding spots in the skin and mucous membrane, or hemorrhagic tendency (find themselves prone to bleeding in gums, nose, skin and mucous membrane, or hemoptysis); (5) History of active bleeding (such as peptic ulcer, hemorrhoids, active tuberculosis, subacute bacterial endocarditis, etc.); (6) Blood platelet count less than 150 × 10^9^/L; (7) Trauma history (e.g., craniocerebral trauma) recently; (8) Past history of unexplained syncope or convulsion; (9) History of organic or psychogenic disease or the disabled; (10) Persons who were unlikely to participate in the study (such as the infirm) in the investigator's opinion; (11) Have donated blood or experienced blood collection in other trials within 3 months^75^.

Once enrolled, subjects were randomly assigned to 11 groups (Fig. 4). Groups 1 and 2 were designed to identify the appropriate dose and contained only 2 subjects each, and groups 3–11 contained 6 or 12 subjects. In groups 1–8, single dose of anfibatide was injected as a bolus *i.v.* push based on a modified Fibonacci method at: 0.33, 0.66, 1, 1.5, 2, 3, 4, and 5 µg/60 kg body weight (Fig. 4, Part A; single dose groups). In groups 9–11, the first dose of anfibatide was given as a slow *i.v.* push (3, 3, or 5 µg/60 kg) followed by a continuous infusion at a constant rate of 0.12 μg/60 kg/h for 24 h (Fig. 4, Part B; multiple dose groups). In group 9, the infusion began at 1.5 h after the bolus.

The primary outcome of the trial was the frequency and severity of adverse events. A serious adverse event was defined as any untoward medical occurrence that the subject experienced while involved in the study that may or may not have a causal relationship with anfibatide and that resulted in death, was life-threatening, required inpatient hospitalization, or resulted in persistent or significant disability. We also measured hemostatic parameters, including platelet count, bleeding time, coagulation, and ex vivo platelet aggregation.

### Drug administration and tolerance test

Anfibatide was infused *i.v.* over 5 min. The tolerance test involved a dose escalation paradigm. The study was an open label dose escalation trial, starting with 0.33 μg/60 kg body weight. Each subject received only one dose. After completion of each dose, a safety board discussed all findings before the next higher dose could be administered.

### Blood sample collection, platelet counts, and coagulation

Blood samples were collected from subjects’ superficial elbow veins into: anticoagulant tubes (3.8% sodium citrate; BD Vacutainer) for testing platelet aggregation and blood coagulation, and dry tubes without any anticoagulant for ELISA and coagulation time assay. In Part A (Group 3–8), samples were collected at 0, 5, 15, 30 min, 1, 2, 3, 4, 5, 6, 8, 12, 24, and 48 h after drug administration. In Part B (Group 9–11), samples were collected at 0, 5 min and 1.5 h (1.5 h was for Group 9 only) after loading dose, and 1, 3, 6, 12, 18, 24, 25, 26, 28, 36, and 48 h after constant rate infusion. One tube was immediately used for hematology and blood chemistry tests. Samples were analyzed with a Stago Compact CT Blood coagulation analyzer (Stago, France) for thrombin time, prothrombin time, activated partial thromboplastin time, international normalized ratio, and d-dimer. From the other tubes, PRP was used for platelet aggregation. Platelet counts were analyzed using a Hamecell Plus blood analyzer (Biocode Hycel, France). Finally, 1 mL of blood was incubated at 37 ºC; every 30 s the tube was tilted to observe blood fluidity until complete coagulation occurred.

### Bleeding time

Bleeding time for single dose groups 3–8 were measured at 0 min, 5 min, 30 min, 2 h, 4 h, and 6 h, while for multiple dose groups 9–11 were measured at 0 min, 5 min, 3 h, 12 h, 24 h, and 26 h. A blood pressure cuff on the volunteer’s upper arm was inflated to 40 mmHg. An incision making device, Surgicutt (ITC, USA) device, was placed on the forearm and used to make a standard-sized cut (width 5 mm, depth 1 mm, avoiding veins) as per manufacturer’s instructions. Filter paper was used to draw off blood at 30 s intervals until bleeding stopped completely. The time when the incision was made until all bleeding stopped was measured. The bleeding time was determined to the nearest 30 s, as per manufacturer’s instructions.

### Platelet aggregation in PRP from anfibatide-treated participants

Platelet aggregation assays were performed using ristocetin (Applied BioPhysics, USA), which induces GPIbα receptor-dependent platelet aggregation, as described^9,21,80^. Ristocetin (15 µL, 60 mg/mL in saline) was added to 350 µL PRP from anfibatide-treated volunteers in colorimetrical cylinders and incubated at 37 °C. Aggregation was analyzed using a LBY-NJ4 platelet aggregometer (Beijing Precil, China) and curves were read within 5 min. The laboratory used standardized protocols and instructions provided by the manufacturer.

The rate of inhibition of platelet aggregation at different time points was calculated as follows: % Inhibition of platelet aggregation (Time n) = (% of platelet aggregation (Time 0)−% of platelet aggregation (Time n))/% of platelet aggregation (Time 0). Note that Time 0 means before drug infusion; Time “n” means n min/h after drug infusion.

### Safety and adverse events

Drug safety was monitored via standard clinical laboratory parameters. Adverse events were monitored during the entire course of the study through investigator inquiries, spontaneous reports, and clinical evaluations including physical examinations, vital sign measurement, electrocardiography, and clinical laboratory tests (e.g. hematology, blood chemistry, coagulation, and urinalysis). A detailed description is provided in the Supplementary Tables 10 to 18.

### Urinalysis

Urine samples were collected and analyzed before, and 24 h after drug administration. All urine was assayed using an automatic urine analyzer SC7KBXL (Kobold, Hofheim, Germany).

### Anti-anfibatide antibodies

To check whether anfibatide induces generation of anti-anfibatide antibodies, serum samples collected 1 month after drug administration were analyzed by ELISA at Zhaoke Pharmaceutical Limited (China) according to standard laboratory practices.

### Statistical analysis

Data are presented by summary statistics as follows: number of observations, arithmetic mean, and SD or SEM as indicated. For the in vitro data, ANOVA was used to test for significant differences between multiple groups; unpaired, two-tailed Student’s t-test was used to test for significant differences between 2 groups. All groups in clinical trial I were normally distributed as determined by the Kolmogorov–Smirnov test; therefore, ANOVA and Bonferroni analysis were used to test for significant differences between multiple dosing groups; unpaired, two-tailed Student’s t-test was used to test for significant differences between 2 groups. Repeated measures ANOVA was used to assess differences in aggregation after anfibatide administration. All adverse events were recorded with: type, onset, duration, severity, and drug relation. Vital signs (blood pressure and pulse rate), electrocardiogram, safety laboratory data, and antibody titers were evaluated using descriptive statistics.

### Statement of prior presentation

Some data were orally presented at the XXVI Congress of International Society of Thrombosis and Haemostasis, Amsterdam, Netherlands, July 2013; Annual Meeting of the American Society of Hematology (ASH) in New Orleans, USA, December 2013; and XXVII Congress of International Society on Thrombosis and Haemostasis, Toronto, Canada, June 20, 2015. This study has also been selected for inclusion in the press program of the 55th ASH Annual Meeting.

## Supplementary Information

Supplementary Information.

## Data Availability

All data generated or analyzed during this study are included in this article (and its supplementary information files) and are available from the authors upon reasonable request.
