# Supplementary material for: In vitro assessment and phase I randomized clinical trial of anfibatide a snake venom derived anti-thrombotic agent targeting human platelet GPIbα
Source: Sci Rep. 2021 Jun 3;11:11663. doi: 10.1038/s41598-021-91165-8 (PMC8175443; doi:10.1038/s41598-021-91165-8)
Supplement: Supplementary file 1 — Supplementary Information. [file 41598_2021_91165_MOESM1_ESM.docx]

***In vitro* assessment and phase I** **randomized clinical trial of anfibatide**

**a snake venom derived anti-thrombotic agent targeting human platelet GPIbα**

*Running title: Anfibatide and anti-thrombotic therapy*

**Benjamin Xiaoyi Li,^1,2^* Xiangrong Dai,^1,2^ Xiaohong Ruby Xu,^3-5^** **Reheman Adili,^3,4^ Miguel Antonio Dias Neves,^3-6^ Xi Lei,^3,4^ Chuanbin Shen^3-5^, Guangheng Zhu,^3,4^ Yiming Wang,^3-6^ Hui Zhou,^3,4^ Yan Hou,^3,4^ Tiffany Ni,^3-5^ Yfke Pasman,^3-6^ Zhongqiang Yang,^2^ Fang Qian,^2^ Yanan Zhao,^7^ Yongxiang Gao,^8^ Jing Liu,^8^ Maikun Teng,^8^ Alexandra H. Marshall,^3,4^**  **Eric G. Cerenzia,^3,4,9^**  **Mandy Lokyee Li,^1^ Heyu Ni,^3-6, 9,10^***

^1^Lee’s Pharmaceutical Holdings Limited, Shatin, Hong Kong, China; ^2^Zhaoke Pharmaceutical Co. Limited, Hefei, China; ^3^Department of Laboratory Medicine, Keenan Research Centre for Biomedical Science, Li Ka Shing Knowledge Institute, St. Michael's Hospital, Unity Health Toronto; ^4^Toronto Platelet Immunobiology Group, Toronto, Canada; ^5^Department of Laboratory Medicine and Pathobiology, University of Toronto, Toronto, Canada; ^6^Canadian Blood Services Centre for Innovation, Toronto, Canada; ^7^Wannan Medical College First Affiliated Hospital, Yijishan Hospital, Wuhu, China; ^8^School of Life Sciences, University of Science and Technology of China; ^9^Department of Physiology, and ^10^Department of Medicine, University of Toronto, Toronto, Canada

*Corresponding authors:

**Dr. Benjamin Xiaoyi Li, Ph.D**

1/F, Building 20E, Phase 3, Hong Kong Science Park, Shatin, N.T. Hong Kong SAR. Tel: 852-2314 1282, Fax: 852-2314 1708, Email:drli@leespharm.com.

**Dr. Heyu Ni, MD; Ph.D**

St. Michael’s Hospital, Room 421, LKSKI - Keenan Research Centre, 209 Victoria Street, Toronto, Ontario, CANADA   M5B 1W8. Tel: 1-416-847-1738, Email: Heyu.Ni@unityhealth.to.

**Supplemental Methods**

**1. Purification of Anfibatide**

Prior to the purification process, a crude extract of anfibatide was prepared by weighing 10 g of the *Agkistrodon acutus* snake venom and dissolving with 80 mL of 0.02 mol/L Tris-HCl buffer (pH 8.0). The solution was stored at 4°C overnight. Centrifuge twice at 4000 rpm for 10 min and collected the supernatant. Anfibatide was purified from crude venom through four chromatography steps, with details as follows:

**(i) Anion-exchange Chromatography (DEAE-Sepharose** **Fast Flow Chromatography)**

The crude venom was loaded into a pre-equilibrated DEAE-Sepharose Fast Flow column (XK10.0×60cm). The column was then washed with 0.02 mol/L Tris-HCl (pH 8.0) at 16 mL/min for 6 hours. Subsequently, a typical gradient elution process was performed at a flow rate of 14 mL/min over 24 hours. During the gradient elution process, the NaCl concentration was increased gradually (in a linear fashion) from 0.05 mol/L to 0.5 mol/L with a pH range from 8.0 to 6.0. The eluted fractions were collected by fraction collectors at 35 tubes/hour with each tube containing 22 mL. A total of 1200 mL sample were collected with 800 mg of protein contained. The collected fractions were then passed through an ultrafiltration membrane with molecular-mass cutoff of 5000 kDa for concentration and desalination using Minitan II System (Millipore Corp., Massachusetts).

**(ii) Cation-exchange Chromatography (CM-Sepharose** **Fast Flow Chromatography)**

After the ultrafiltration process, the fractions were centrifuged at 3500 rpm for 10 min to gain the supernatant. The supernatant was loaded into a pre-equilibrated CM-Sepharose Fast Flow column (XK5.0×60cm). The column was then washed with 0.02 mol/L Tris-HCl (pH 6.0) at a flow rate of 5.5 mL/min for 4 hours. Then, gradient elution process was performed with a NaCl concentration gradients (in a linear fashion) from 0 mol/L to 0.5 mol/L and a pH range from 6.0 to 8.0 over 24 hours. The eluted fractions were collected by fraction collectors at 11 tubes/hour with each tube containing 22mL. The second of three peaks of the elution curve was represented as anfibatide peak. A total of 600 mL purified anfibatide were collected, containing 160 mg protein. The collected fractions were then passed through an ultrafiltration membrane with molecular-mass cutoff of 5000 kDa for concentration and desalination using Minitan II System (Millipore Corp., Massachusetts).

**(iii) Monoclonal Antibody-based Affinity Chromatography**

After the ultrafiltration process, the fractions were centrifuged at 3500 rpm for 10min to obtain the supernatant. The supernatant was loaded into a pre-equilibrated monoclonal antibody-based affinity column (XK2.6×40cm, GenScript (Nanjing) Co., Ltd.). The column was then washed with 0.02 mol/L Tris-HCl pH 7.6, 0.15 mol/L NaCl at a slow flow rate of 0.5 mL/min for 5 hours. The proteins were eluted with 0.2 mol/L glycine (pH 2.0) at a flow rate of 2 mL/min for 16 hours. The eluted fractions were collected by an automated fraction collector of AKTA prime plus (GE Healthcare). A total of 16 mg purified anfibatide were collected. The collected fractions were transferred into a dialysis bag and concentrated to approximately 10 mL with fan.

**(iv) Gel Filtration Chromatography (Sephacryl S-100 HR Chromatography)**

The concentrated sample was then loaded into a pre-equilibrated Sephacryl S-100 HR gel filtration column (XK2.6×100cm). The column was then eluted with 0.02 mol/L Tris-HCl pH 7.6, 0.15 mol/L NaCl at a 0.8 mL/min. The eluted fractions were collected by fraction collectors at 30 tubes/hour with each tube containing 1.6 mL. The result showed that gel filtration chromatography elution curve for a single peak, explain for homogeneous component of anfibatide. Purified anfibatide was further evaluated by the SDS-PAGE electrophoresis, which showed that its purity was above 95%.

**2. Enzyme-linked Immunosorbent Assay (ELISA) for the Detection of Anfibatide Concentration in Phase I Clinical Trial**

The ELISA was performed in a 96-well microtiter plate (Nunc). Before adding to wells, the anti-mouse monoclonal anti-agkisacutacin antibody (Genscript, Nanjing, China; cat. no. 20090430; prepared as described by Su *et al.* [1]) was diluted by 1:1000 with the coating buffer (0.05 moI/L NaHCO3, pH 9.6) to produce a concentration of 2.941 μg/mL as the working solution. Wells of plate were sensitized with 100 μL/well of anti- mouse monoclonal anti-agkisacutacin antibody working solution overnight at 4°C. Subsequently, three washes were performed with phosphate-buffered saline with 0.05% Tween 20 (PBS-T, as the washing buffer). The wells were then filled with blocking buffer (1% BSA), and maintained for 2 hours at 37°C. Next, three washes were performed with the washing buffer. Then, 100 μL of series concentration gradient of anfibatide standards solution, or test sample and quality control samples (0.75, 0.15 and 0.025 ng/mL) were added to the wells and incubated at 37°C for 1 hour, with three washes step followed. 100 µL of rabbit polyclonal anti-agkisacutacin antibody (developed in rabbit at the Tiancheng biological technology, Beijing, China; cat. no. 20090508, as described by Zhao Y. N. *et al.* [2]) working solution (diluted from initial concentration of 8.557 mg/mL to 1:425 using the blocking buffer) was added to the appropriate wells and incubated at 37°C for 1 hour. Then, wash the plate with washing buffer three times. 100 μL of horseradish peroxidase (HRP) conjugated Goat-Anti-Rabbit antibody((Pierce, diluted 1:2000 with blocking buffer) were added to the appropriate wells and incubated at 37°C for 1 hour. After incubation, three washes were performed with the washing buffer and 100 µL of the freshly prepared OPD substrate working solution was added to each well. The plate was then placed in dark for 2-5 minutes to allow colour development. The colour reaction was stopped by the addition of 100 µl/well of 1M H2SO4. The spectrophotometric reading was performed at 492 nm.

**References:**

[1] L.J. Su, L. Xiao, T. Zhan, L. Chen, Y.Q. Dong, L. Fang, L.S. Cheng, J. Liu, Preparation and identification of monoclonal antibodies against snake venom C-type lectin like protein Agkisacutacin, Chin. J. Cell. Mol. Immunol. 23 (2007) 1031–1033.

[2] Zhao Y.N. Dai XR, Liu JJ, et al. An indirect sandwich ELISA for the determination of agkisacutacin in human serum: application to pharmacokinetic study in Chinese healthy volunteers. J Pharm Biomed Anal. 2012;70:396-400. doi:10.1016/j.jpba.2012.06.001

1. **Supplementary Tables**

|  | **Index** | **Page No.** |
| --- | --- | --- |
| **Supplementary Table 1.** | **Baseline Characteristics of the Single Dose Groups** | **7** |
| **Supplementary Table 2.** | **Baseline Characteristics of the Multiple Dose Groups** | **7** |
| **Supplementary Table 3.** | **Pharmacokinetic Parameters of Group 9 after Multiple Intravenous Administration of Anfibatide** | **8** |
| **Supplementary Table 4.** | **Pharmacokinetic Parameters of Group 10 after Multiple Intravenous Administration of Anfibatide** | **8** |
| **Supplementary Table 5.** | **Pharmacokinetic Parameters of Group 11 after Multiple Intravenous Administration of Anfibatide** | **8** |
| **Supplementary Table 6.** | **Pharmacodynamic Parameters for the Assessment of Inhibition of Anfibatide on Platelet Aggregation in Healthy Volunteers after Single Intravenous Bolus Injection at Dose Levels of 1, 1.5, 2, 3, 4 and 5 µg/60kg, Respectively** | **9** |
| **Supplementary Table 7.** | **Pharmacodynamic Parameters for the Assessment of Inhibition of Anfibatide on Platelet Aggregation in Group 9 after Multiple Intravenous Administration of Anfibatide** | **10** |
| **Supplementary Table 8.** | **Pharmacodynamic Parameters for the Assessment of Inhibition of Anfibatide on Platelet Aggregation in Group 10 after Multiple Intravenous Administration of Anfibatide** | **10** |
| **Supplementary Table 9.** | **Pharmacodynamic Parameters for the Assessment of Inhibition of Anfibatide on Platelet Aggregation in Group 11 after Multiple Intravenous Administration of Anfibatide** | **10** |

| **Supplementary Table 10.** | | **Vital Signs of the Single Dose Groups and Multiple Dose Groups at Baseline** | **11** |
| --- | --- | --- | --- |
| **Supplementary Table 11.** | **Vital Signs of the Single Dose Groups and Multiple Dose Groups after Treatment of Anfibatide** | | **11** |
| **Supplementary Table 12.** | **Laboratory Parameters of the 0.33 μg and 0.66 μg Dosing Groups before and after Treatment of Anfibatide (Mean ± SD)** | | **12** |
| **Supplementary Table 13.** | **Laboratory Parameters of the 1.0 μg and 1.5 μg Dosing Groups before and after Treatment of Anfibatide (Mean ± SD)** | | **13** |
| **Supplementary Table 14.** | **Laboratory Parameters of the 2.0 μg and 3.0 μg Dosing Groups before and after Treatment of Anfibatide (Mean ± SD)** | | **14** |
| **Supplementary Table 15.** | **Laboratory Parameters of the 4.0 μg and 5.0 μg Dosing Groups before and after Treatment of Anfibatide (Mean ± SD)** | | **15** |
| **Supplementary Table 16.** | **Laboratory Abnormalities of the Single Dose Groups after Treatment of Anfibatide** | | **16** |
| **Supplementary Table 17.** | **Laboratory Parameters of the Multiple Dose Groups before and after Treatment of Anfibatide (Mean ± SD)** | | **17** |
| **Supplementary Table 18.** | **Laboratory Abnormalities of the Multiple Dose Groups after Treatment of Anfibatide** | | **18** |

## Supplementary Table 1. Baseline Characteristics of the Single Dose Groups

##

| Group | Age | Height | Weight | BMI |
| --- | --- | --- | --- | --- |
|  | (years) | (m) | (kg) | (kg/m^2^) |
| 1 | 23.0±0.0 | 1.66±0.06 | 61.5±0.71 | 22.37±1.78 |
| 2 | 22.0±1.41 | 1.67±0.05 | 59±4.24 | 21.27±0.27 |
| 3 | 21.8±1.75 | 1.68±0.08 | 59.15±8.33 | 20.97±1.88 |
| 4 | 21.20±1.14 | 1.71±0.09 | 58.50±10.75 | 19.96±1.99 |
| 5 | 21.10±1.52 | 1.67±0.07 | 58.50±7.26 | 20.83±1.36 |
| 6 | 21.89±1.36 | 1.70±0.09 | 59.78±8.91 | 20.58±1.80 |
| 7 | 22.30±2.00 | 1.67±0.10 | 56.15±7.48 | 20.11±1.18 |
| 8 | 21.20±1.23 | 1.69±0.07 | 58.30±5.40 | 20.41±1.34 |

Abbreviation: **BMI =** Body Mass Index.

## Supplementary Table 2. Baseline Characteristics of the Multiple Dose Groups

| Group | Age | Height | Weight | BMI |
| --- | --- | --- | --- | --- |
|  | (years) | (m) | (kg) | (kg/m^2^) |
| 9 | 22.67±1.03 | 1.73±0.04 | 65.33±6.15 | 22.15±2.72 |
| 10 | 21.75±1.66 | 1.66±0.07 | 57.83±6.81 | 21.05±1.23 |
| 11 | 21.83±1.27 | 1.66±0.11 | 58.67±9.18 | 20.99±1.58 |

Abbreviation: **BMI =** Body Mass Index.

## Supplementary Table 3. Pharmacokinetic Parameters of Group 9 after Multiple Intravenous Administration of Anfibatide

| Parameter | Unit | 9A | 9B | 9C | 9D | 9E | 9F | Mean |
| --- | --- | --- | --- | --- | --- | --- | --- | --- |
| Cmax | ng/mL | 0.414 | 0.36 | 0.429 | 0.487 | 0.472 | 0.513 | 0.446±0.056 |
| Tmax | h | 29.5 | 29.5 | 29.5 | 29.5 | 29.5 | 29.5 | 29.5 |
| AUC(0-t) | ng·h/mL | 8.4 | 7.92 | 8.47 | 8.13 | 9.17 | 8.98 | 8.51±0.48 |

Abbreviations: **AUC(0-t)** = area under the plasma concentration-time curve from time 0 to time t; **Cmax =** maximum drug concentration; Tmax = time to maximum concentration.

## Supplementary Table 4. Pharmacokinetic Parameters of Group 10 after Multiple Intravenous Administration of Anfibatide

| Parameter | Unit | 10C | 10D | 10E | 10F | 10G | 10H | 10I | 10J | 10K | 10L | Mean |
| --- | --- | --- | --- | --- | --- | --- | --- | --- | --- | --- | --- | --- |
| Cmax | ng/mL | 0.522 | 0.476 | 0.443 | 0.484 | 0.500 | 0.484 | 0.485 | 0.459 | 0.496 | 0.476 | 0.482±0.022 |
| Tmax | h | 28 | 28 | 28 | 28 | 28 | 28 | 28 | 28 | 28 | 28 | 28 |
| AUC(0-t) | ng·h/mL | 6.04 | 5.59 | 5.97 | 5.97 | 6.20 | 5.06 | 6.02 | 5.73 | 5.66 | 6.18 | 5.84±0.35 |

Abbreviations: **AUC(0-t)** = area under the plasma concentration-time curve from time 0 to time t; **Cmax =** maximum drug concentration; Tmax = time to maximum concentration.

## Supplementary Table 5. Pharmacokinetic Parameters of Group 11 after Multiple Intravenous Administration of Anfibatide

| Parameter | Unit | 11A | 11B | 11C | 11D | 11E | 11F | 11G | 11H | 11I | 11J | 11K | 11L | Mean |
| --- | --- | --- | --- | --- | --- | --- | --- | --- | --- | --- | --- | --- | --- | --- |
| Cmax | ng/mL | 0.691 | 0.76 | 0.717 | 0.7 | 0.774 | 0.629 | 0.695 | 0.645 | 0.721 | 0.605 | 0.628 | 0.734 | 0.691±0.054 |
| Tmax | h | 28 | 28 | 28 | 28 | 28 | 28 | 28 | 28 | 28 | 28 | 28 | 28 | 28 |
| AUC(0-t) | ng·h/mL | 9.21 | 10.05 | 10.33 | 10.58 | 10.62 | 9.76 | 8.64 | 9.49 | 10.47 | 9.04 | 11.01 | 10.52 | 9.98±0.74 |

Abbreviations: **AUC(0-t)** = area under the plasma concentration-time curve from time 0 to time t; **Cmax =** maximum drug concentration; Tmax = time to maximum concentration.

## Supplementary Table 6. Pharmacodynamic Parameters for the Assessment of Inhibition of Anfibatide on Platelet Aggregation in Healthy Volunteers after Single Intravenous Bolus Injection at Dose Levels of 1, 1.5, 2, 3, 4 and 5 µg/60kg, Respectively

| Parameter |  |  |  |  | **1 µg/kg Dosing Group** | |  |  |  |  |
| --- | --- | --- | --- | --- | --- | --- | --- | --- | --- | --- |
|  | 3A | 3B | 3C | 3D | 3E | 3F | 3H | 3I | 3J | 3K |
| Emax (%) | 100.0 | 80.8 | 90.1 | 66.2 | 85.7 | 100.0 | 61.8 | 64.3 | 75.8 | 70.4 |
| Tmax (h) | 0.5 | 0.5 | 0.085 | 0.25 | 0.085 | 0.25 | 0.085 | 0.085 | 0.085 | 0.085 |
| Tmin (h) | 6 | 6 | 6 | 6 | 3 | 3 | 3 | 4 | 3 | 4 |
| AUEC | 332.6 | 222.6 | 173.4 | 131.1 | 53.4 | 176.1 | 89.9 | 68.5 | 106.1 | 140.0 |
| **1.5 µg/kg Dosing Group** | | | | | | | | | | |
|  | 4A | 4B | 4C | 4D | 4E | 4F | 4G | 4H | 4I | 4J |
| Emax (%) | 71.7 | 70.6 | 100.0 | 97.0 | 97.4 | 98.1 | 59.7 | 60.3 | 87.5 | 64.7 |
| Tmax (h) | 0.085 | 0.085 | 0.085 | 1 | 0.085 | 0.085 | 0.085 | 0.085 | 0.085 | 0.085 |
| Tmin (h) | 5 | 6 | 4 | 4 | 5 | 5 | 4 | 5 | 3 | 5 |
| AUEC | 173.7 | 167.3 | 210.1 | 244.0 | 267.6 | 277.2 | 78.7 | 171.2 | 114.4 | 129.4 |
| **2 µg/kg Dosing Group** | | | | | | | | | | |
|  | 5A | 5B | 5C | 5D | 5E | 5F | 5G | 5H | 5I | 5J |
| Emax (%) | 91.5 | 91.3 | 86.5 | 85.7 | 96.5 | 100.0 | 88.6 | 99.3 | 100.0 | 59.9 |
| Tmax (h) | 0.085 | 0.085 | 0.085 | 0.085 | 0.085 | 0.085 | 0.085 | 0.085 | 0.085 | 0.085 |
| Tmin (h) | 6 | 6 | 6 | 6 | 5 | 6 | 6 | 6 | 6 | 6 |
| AUEC | 235.4 | 258.6 | 262.6 | 303.7 | 328.0 | 273.5 | 223.8 | 198.5 | 139.0 | 91.7 |
| **3 µg/kg Dosing Group** | | | | | | | | | | |
|  | 6A | 6B | 6C | 6D | 6E | 6F | 6G | 6H | 6I | 6J |
| Emax (%) | 98.1 | 98.6 | 98.5 | 98.1 | 83.4 | 98.1 | 96.2 | 86.4 | 73.6 | Drop out |
| Tmax (h) | 0.085 | 0.5 | 0.085 | 0.085 | 0.085 | 0.085 | 0.085 | 0.085 | 0.085 |  |
| Tmin (h) | 8 | 8 | 6 | 8 | 6 | 8 | 8 | 6 | 6 |  |
| AUEC | 361.8 | 351.1 | 333.8 | 382.8 | 239.2 | 300.3 | 331.9 | 182.2 | 214.9 |  |
| **4 µg/kg Dosing Group** | | | | | | | | | | |
|  | 7A | 7B | 7C | 7D | 7E | 7F | 7G | 7H | 7I | 7J |
| Emax (%) | 99.1 | 94.8 | 97.8 | 100.0 | 94.7 | 98.5 | 98.6 | 100.0 | 96.5 | 98.5 |
| Tmax (h) | 0.085 | 0.5 | 0.085 | 0.5 | 0.085 | 0.5 | 0.085 | 0.085 | 0.085 | 0.085 |
| Tmin (h) | 8 | 8 | 8 | 8 | 8 | 8 | 8 | 8 | 8 | 8 |
| AUEC | 439.1 | 447.2 | 446.5 | 408.0 | 338.1 | 338.7 | 244.7 | 283.5 | 271.0 | 172.7 |
| **5 µg/kg Dosing Group** | | | | | | | | | | |
|  | 8A | 8B | 8C | 8D | 8E | 8F | 8G | 8H | 8I | 8J |
| Emax (%) | 100.0 | 99.1 | 97.7 | 94.0 | 96.2 | 93.0 | 96.7 | 93.6 | 98.5 | 98.1 |
| Tmax (h) | 0.085 | 0.5 | 0.085 | 0.5 | 0.085 | 0.5 | 0.085 | 0.085 | 0.085 | 0.085 |
| Tmin (h) | 8 | 8 | 8 | 8 | 8 | 8 | 8 | 8 | 8 | 8 |
| AUEC | 396.9 | 421.0 | 419.2 | 362.9 | 226.5 | 275.2 | 215.4 | 286.7 | 208.9 | 426.8 |

Abbreviations: **AUEC** = area under the effect curve; **Emax** = maximal inhibitory effect on platelet aggregation; **Tmax** = time to Emax; **Tmin** =time of minimal inhibitory effect on platelet aggregation.

## Supplementary Table 7. Pharmacodynamic Parameters for the Assessment of Inhibition of Anfibatide on Platelet Aggregation in Group 9 after Multiple Intravenous Administration of Anfibatide

| Parameter | Unit | 9A | 9B | 9C | 9D | 9E | 9F | Mean |
| --- | --- | --- | --- | --- | --- | --- | --- | --- |
| Emax | % | 69.5 | 60.5 | 100 | 95.8 | 97.7 | 64.5 | 81.3±18.4 |
| Tmax,E | H | 0.083 | 0.083 | 2.5 | 0.083 | 0.083 | 7.5 | 1.72±2.99 |
| AUEC | %·h | 962.9 | 1179.6 | 1453.4 | 1486.5 | 1419.4 | 990.6 | 1248.7±236.9 |

Abbreviations: **AUEC** = area under the effect curve; **Emax** = maximal inhibitory effect on platelet aggregation; **Tmax,E** = time to Emax.

## Supplementary Table 8. Pharmacodynamic Parameters for the Assessment of Inhibition of Anfibatide on Platelet Aggregation in Group 10 after Multiple Intravenous Administration of Anfibatide

| Parameter | Unit | 10A | 10B | 10C | 10D | 10E | 10F | 10G | 10H | 10I | 10J | 10K | 10L | Mean |
| --- | --- | --- | --- | --- | --- | --- | --- | --- | --- | --- | --- | --- | --- | --- |
| Emax | % | 81.5 | 87.1 | 83 | 73.6 | 86.7 | 92.4 | 74.2 | 74 | 93.5 | 83.4 | 83.8 | 73.8 | 82.2±7.1 |
| Tmax,E | H | 0.083 | 0.083 | 0.083 | 6 | 12 | 0.083 | 6 | 0.083 | 12 | 24 | 18 | 18 | 8.03±8.58 |
| AUEC | %·h | 1775.2 | 1925.0 | 1961.8 | 1575.7 | 1859.9 | 2017.3 | 1470.9 | 1344.1 | 1669.3 | 1559.6 | 1674.6 | 1499.2 | 1694.4±214.7 |

Abbreviations: **AUEC** = area under the effect curve; **Emax** = maximal inhibitory effect on platelet aggregation; **Tmax,E** = time to Emax.

## Supplementary Table 9. Pharmacodynamic Parameters for the Assessment of Inhibition of Anfibatide on Platelet Aggregation in Group 11 after Multiple Intravenous Administration of Anfibatide

| Parameter | Unit | 11A | 11B | 11C | 11D | 11E | 11F | 11G | 11H | 11I | 11J | 11K | 11L | Mean |
| --- | --- | --- | --- | --- | --- | --- | --- | --- | --- | --- | --- | --- | --- | --- |
| Emax | % | 100 | 100 | 97.7 | 89.1 | 100 | 99.9 | 98.7 | 79.6 | 97.5 | 85.9 | 96.8 | 93.6 | 94.9±6.7 |
| Tmax,E | h | 0.083 | 24 | 6 | 6 | 24 | 0.083 | 6 | 0.083 | 1 | 1 | 12 | 0.083 | 6.69±8.88 |
| AUEC | %·h | 2540.9 | 2491.1 | 2196 | 1669.1 | 2519.5 | 2543 | 2287.1 | 1788.7 | 2054.3 | 2094.2 | 2215.8 | 1881.9 | 2190.1±303.3 |

Abbreviations: **AUEC** = area under the effect curve; **Emax** = maximal inhibitory effect on platelet aggregation; **Tmax,E** = time to Emax.

## Supplementary Table 10. Vital Signs of the Single Dose Groups and Multiple Dose Groups at Baseline

| Group | Body Temperature | Heart Rate | Respiratory Rate | Blood Pressure |
| --- | --- | --- | --- | --- |
|  | (℃) | (beat/min) | (rate/min) | (mmHg) |
| 1 | 36.75±0.35 | 60.0±5.66 | 18±0 | 100±0 / 71±7 |
| 2 | 36.2±0 | 70±14.14 | 20±0 | 108±11 / 70±0 |
| 3 | 36.42±0.34 | 70.40±9.83 | 19.60±1.84 | 107±7 / 68±9 |
| 4 | 36.33±0.19 | 74.20±11.64 | 19.10±0.88 | 113±5 / 73±4 |
| 5 | 36.40±0.32 | 71.10±8.85 | 18.30±1.77 | 114±8 / 71±6 |
| 6 | 36.32±0.31 | 70.44±11.11 | 17.67±2.45 | 107±11 / 73±7 |
| 7 | 36.47±0.26 | 75.20±11.30 | 17.60±1.78 | 110±10 / 72±7 |
| 8  9  10  11 | 36.50±0.24  36.47±0.33  36.39±0.29  36.38±0.15 | 66.20±7.55  69.83±8.28  70.33±10.79  76.92±10.21 | 18.30±1.16  18.17±1.17  18.50±0.67  18.75±0.62 | 112±6 / 75±4  109±9 / 74±5  111±11 / 74±8  115±10 / 78±7 |

**Supplementary Table 11. Vital Signs of the Single Dose Groups and Multiple Dose Groups after Treatment of Anfibatide**

| Group | Body Temperature | Heart Rate | Respiratory Rate | Blood Pressure |
| --- | --- | --- | --- | --- |
|  | (℃) | (beat/min) | (rate/min) | (mmHg) |
| 1 | 36.5±0.0 | 64.0±8.49 | 19.5±0.71 | 105±7 / 70±0 |
| 2 | 36.1±0.14 | 59.5±2.12 | 19.5±0.71 | 105±7 / 62±3 |
| 3 | 36.27±0.28 | 70.10±10.77 | 18.70±1.16 | 104±8 / 65±8 |
| 4 | 36.50±0.32 | 73.60±7.59 | 18.20±1.75 | 105±12 / 67±8 |
| 5 | 36.40±0.17 | 67.20±7.30 | 19.00±0.82 | 111±7 / 74±4 |
| 6 | 36.53±0.29 | 69.89±14.95 | 17.56±1.88 | 106±13 / 72±9 |
| 7 | 36.60±0.27 | 75.40±9.79 | 18.40±1.17 | 110±13 / 72±8 |
| 8  9  10  11 | 36.21±0.27  36.33±0.27  36.31±0.16  36.35±0.12 | 68.20±10.41  71.33±10.63  69.33±8.06  72.83±8.73 | 18.40±1.84  19.00±0.63  18.58±0.79  18.92±0.51 | 106±10 / 71±8  108±4 / 72±5  107±8 / 74±5  105±5 / 70±5 |

**Supplementary Table 12. Laboratory Parameters of the 0.33 μg and 0.66 μg Dosing Groups before and after Treatment of Anfibatide (Mean ± SD)**

### 0.33 μg Dosing Group (Group 1) 0.66 μg Dosing Group (Group 2)

|  | Parameter | Unit | Pre-treatment | Post-treatment | Pre-treatment | Post-treatment |
| --- | --- | --- | --- | --- | --- | --- |
|  | RBC | ×10^12^/L | 4.89±0.33 | 4.75±0.06 | 4.86±0.47 | 5.03±0.46 |
|  | WBC | ×10^9^/L | 5.40±1.98 | 6.60±3.39 | 7.70±1.27 | 7.90±0.71 |
| Hematology | PLT | ×10^9^/L | 201.50±12.02 | 207.50±3.54 | 226.00±79.20 | 238.00±94.75 |
|  | HGB | g/L | 164.50±0.71 | 158.00±9.90 | 146.00±5.66 | 152.00±5.66 |
|  | Hct |  | 0.47±0.00 | 0.46±0.02 | 0.44±0.02 | 0.45±0.02 |
|  | TP | g/L | 75.55±2.62 | 72.80±3.82 | 80.00±2.12 | 79.10±3.68 |
|  | ALB | g/L | 51.85±1.63 | 49.30±3.68 | 51.70±2.12 | 51.90±1.98 |
|  | TBIL | umol/L | 18.55±5.59 | 19.65±0.64 | 10.10±2.97 | 12.90±4.24 |
|  | AST | U/L | 22.00±9.90 | 21.50±12.02 | 15.00±2.83 | 14.00±4.24 |
|  | ALT | U/L | Not tested | 19.00±8.49 | Not tested | Not tested |
|  | γ-GT | U/L | 17.50±2.12 | 15.00±1.41 | 44.50±23.33 | 38.00±15.56 |
|  | ALP | U/L | 87.00±41.01 | Not Tested | 98.50±40.31 | 89.50±23.33 |
|  | LDH | U/L | 166.50±28.99 | 173.00±31.11 | 131.00±1.41 | 136.00±9.90 |
|  | Ca^2^＋ | mmol/L | 2.48±0.01 | 2.43±0.06 | 2.59±0.13 | 2.49±0.07 |
| Serum | P | mmol/L | 1.05±0.32 | 1.14±0.26 | 1.34±0.08 | 1.28±0.21 |
| Chemistry | K^＋^ | mmol/L | 4.35±0.35 | 3.95±0.35 | 4.00±0.00 | 3.80±0.00 |
| Na＋ | | mmol/L | 137.50±2.12 | 139.00±1.41 | 139.50±0.71 | 140.00±0.00 |
| BUN | | mmol/L | 5.15±2.33 | 5.25±1.34 | 4.70±0.85 | 5.90±0.14 |
| Cr | | umol/L | 83.50±2.12 | 89.50±9.19 | 92.00±1.41 | 93.00±1.41 |
| URIC | | umol/L | 294.00±18.38 | 301.00±38.18 | 323.0±4.24 | 315.50±2.12 |
| Glu | | mmol/L | 5.40±0.48 | 4.79±0.22 | 5.33±0.03 | 4.97±0.25 |
| TC | | mmol/L | 4.00±0.81 | 4.10±0.93 | 4.26±0.55 | 4.37±0.55 |
| TG | | mmol/L | 0.74±0.04 | 1.04±0.45 | 1.07±0.08 | 1.30±0.08 |
| LPS | | U/L | 58.50±26.16 | 30.50±2.12 | 33.50±16.26 | 34.00±18.38 |
| AMY | | U/L | 59.50±17.68 | 61.00±21.21 | 77.00±18.38 | 89.50±53.03 |
| HR | | beat/min | 54.50±12.02 | 61.50±12.02 | 66.00±8.49 | 59.00±2.83 |
| ECG PR  QRS | | msec  msec | 157.00±16.97  112.50±9.19 | 157.00±5.66  107.00±2.83 | 156.50±19.09  107.50±14.85 | 159.50±23.33  111.50±14.85 |
| QTc | | msec | 379.00±48.08 | 380.50±31.82 | 400.50±31.82 | 396.00±18.38 |
| RV5+SV1 | | mV | 2.63±0.49 | 2.98±0.54 | 3.30±0.79 | 3.24±0.88 |
| ST segment | |  | Normal | Normal | Normal | Normal |

Abbreviations: **ALB** = albumin; **ALP** = alkaline phosphatase; **ALT** = alanine aminotransferase; **AMY** = amylase; **AST** = aspartate aminotransferase; **BUN** = blood urea nitrogen; **Ca^2+^** = calcium; **Cr** = creatinine; **ECG** = electrocardiogram; **Glu** = glucose; **Hct** = Hematocrit; **HGB** = Hemoglobin; **HR** = Heart Rate; **γ-GT** =gamma-glutamyltransferase; **K^+^** = potassium; **LDH** = lactate dehydrogenase; **LPS** = lipase; **Na^+^** = Sodium; **P** = phosphorus; **PLT** = Platelet; **PR** = PR interval; **QRS** = QRS Interval; **QTc** = Corrected QT interval; **RBC** = red blood cell; **RV5+SV1** = RV5+SV1 amplitude; **SD** = standard deviation; **TBIL** = total bilirubin; **TC** = total cholesterol; **TG** = triglycerides; **TP** = total protein; **URIC** = Uric acid; **WBC** = White blood cell.

## Supplementary Table 13. Laboratory Parameters of the 1.0 μg and 1.5 μg Dosing Groups before and after Treatment of Anfibatide (Mean ± SD)

### 1.0 μg Dosing Group (Group 3) 1.5 μg Dosing Group (Group 4)

|  | Parameter | Unit | Pre-treatment | Post-treatment | Pre-treatment | Post-treatment |
| --- | --- | --- | --- | --- | --- | --- |
|  | RBC | ×10^12^/L | 4.79±0.41 | 4.70±0.45 | 4.68±0.50 | 4.76±0.53 |
|  | WBC | ×10^9^/L | 6.20±1.18 | 6.36±1.48 | 5.93±1.37 | 6.59±1.62 |
| Hematology | PLT | ×10^9^/L | 280.80±79.29 | 269.40±68.05 | 220.20±69.59 | 218.90±69.01 |
|  | HGB | g/L | 138.80±16.96 | 136.00±17.68 | 135.90±19.88 | 142.90±21.45 |
|  | Hct |  | 0.42±0.05 | 0.41±0.04 | 0.42±0.06 | 0.43±0.06 |
|  | TP | g/L | 78.20±4.38 | 78.10±4.48 | 75.02±3.17 | 77.68±3.66 |
|  | ALB | g/L | 49.59±1.99 | 49.36±2.65 | 47.87±2.25 | 49.29±1.93 |
|  | TBIL | umol/L | 16.53±4.08 | 18.80±9.33 | 19.40±6.85 | 17.32±9.29 |
|  | AST | U/L | 18.20±3.22 | 17.80±3.71 | 19.80±8.94 | 15.00±2.79 |
|  | ALT | U/L | 13.29±8.71 | 15.80±9.17 | 17.80±10.77 | 14.90±7.05 |
|  | γ-GT | U/L | 14.10±5.70 | 13.30±5.70 | 17.50±10.44 | 16.30±9.50 |
|  | ALP | U/L | 72.90±20.47 | 71.60±23.91 | 76.10±15.93 | 67.20±15.62 |
|  | LDH | U/L | 148.00±30.50 | 144.70±64.32 | 138.90±46.35 | 149.90±40.58 |
|  | Ca^2^＋ | mmol/L | 2.47±0.07 | 2.48±0.10 | 2.44±0.10 | 2.33±0.21 |
| Serum | P | mmol/L | 1.13±0.15 | 1.14±0.16 | 1.09±0.10 | 1.19±0.14 |
| Chemistry | K^＋^ | mmol/L | 3.81±0.16 | 3.95±0.27 | 3.77±0.13 | 3.70±0.21 |
| Na＋ | | mmol/L | 138.80±2.66 | 140.00±3.65 | 142.90±2.77 | 139.00±1.05 |
| BUN | | mmol/L | 4.65±1.01 | 4.93±0.96 | 4.48±0.84 | 3.91±0.84 |
| Cr | | umol/L | 84.40±18.01 | 85.10±20.02 | 79.20±23.13 | 79.60±23.25 |
| URIC | | umol/L | 326.50±119.71 | 325.20±137.77 | 318.20±70.15 | 301.40±56.27 |
| Glu | | mmol/L | 4.87±0.44 | 4.85±0.58 | 4.80±0.44 | 4.86±0.51 |
| TC | | mmol/L | 4.04±0.36 | 3.96±0.44 | 3.71±00.50 | 3.82±0.40 |
| TG | | mmol/L | 0.83±0.34 | 0.83±0.29 | 0.84±0.30 | 1.07±0.53 |
| LPS | | U/L | 31.50±10.62 | 36.30±12.08 | 36.00±34.57 | 30.70±16.84 |
| AMY | | U/L | 62.10±17.31 | 62.40±17.22 | 44.30±13.33 | 59.90±22.20 |
| HR | | beat/min | 70.80±11.31 | 70.90±13.32 | 74.90±12.93 | 69.10±9.70 |
| PR | | msec | 144.90±9.89 | 147.70±12.33 | 143.90±26.39 | 147.60±25.05 |
| ECG QRS | | msec | 100.70±10.01 | 101.30±8.91 | 99.40±3.53 | 102.00±8.10 |
| QTc | | msec | 410.80±21.52 | 416.70±25.70 | 422.80±23.93 | 406.50±15.49 |
| RV5+SV1 | | mV | 2.36±0.73 | 2.30±0.94 | 2.08±0.71 | 2.22±0.81 |
| ST segment | |  | Normal | Normal | Normal | Normal |

Abbreviations: **ALB** = albumin; **ALP** = alkaline phosphatase; **ALT** = alanine aminotransferase; **AMY** = amylase; **AST** = aspartate aminotransferase; **BUN** = blood urea nitrogen; **Ca^2+^** = calcium; **Cr** = creatinine; **ECG** = electrocardiogram; **Glu** = glucose; **Hct** = Hematocrit; **HGB** = Hemoglobin; **HR** = Heart Rate; **γ-GT** =gamma-glutamyltransferase; **K^+^** = potassium; **LDH** = lactate dehydrogenase; **LPS** = lipase; **Na^+^** = Sodium; **P** = phosphorus; **PLT** = Platelet; **PR** = PR interval; **QRS** = QRS Interval; **QTc** = Corrected QT interval; **RBC** = red blood cell; **RV5+SV1** = RV5+SV1 amplitude; **SD** = standard deviation; **TBIL** = total bilirubin; **TC** = total cholesterol; **TG** = triglycerides; **TP** = total protein; **URIC** = Uric acid; **WBC** = White blood cell.

## Supplementary Table 14. Laboratory Parameters of the 2.0 μg and 3.0 μg Dosing Groups before and after Treatment of Anfibatide (Mean ± SD)

### 2.0 μg Dosing Group (Group 5) 3.0 μg Dosing Group (Group 6)

|  | Parameter | Unit | Pre-treatment | Post-treatment | Pre-treatment | Post-treatment |
| --- | --- | --- | --- | --- | --- | --- |
|  | RBC | ×10^12^/L | 4.77±0.38 | 4.74±0.47 | 4.72±0.45 | 4.72±0.66 |
|  | WBC | ×10^9^/L | 5.38±0.76 | 6.32±2.12 | 5.99±1.02 | 5.64±0.92 |
| Hematology | PLT | ×10^9^/L | 240.10±49.59 | 256.00±48.05 | 223.00±59.57 | 224.22±48.62 |
|  | HGB | g/L | 146.50±12.31 | 145.90±15.13 | 142.44±12.34 | 142.89±17.95 |
|  | Hct |  | 0.44±0.04 | 0.44±0.05 | 0.43±0.04 | 0.43±0.06 |
|  | TP | g/L | 76.17±4.46 | 76.51±4.23 | 73.89±2.75 | 76.79±5.95 |
|  | ALB | g/L | 48.40±1.49 | 47.21±1.91 | 44.90±1.61 | 47.86±2.76 |
|  | TBIL | umol/L | 19.53±11.22 | 18.48±10.78 | 13.99±5.98 | 14.50±5.30 |
|  | AST | U/L | 20.40±1.84 | 18.10±2.33 | 2.044±5.68 | 18.89±5.21 |
|  | ALT | U/L | 12.50±5.23 | 10.90±3.96 | 17.56±15.81 | 15.56±13.05 |
|  | γ-GT | U/L | 16.20±7.22 | 15.40±6.96 | 14.22±8.41 | 11.56±6.33 |
|  | ALP | U/L | 82.80±28.22 | 77.30±30.28 | 73.78±11.55 | 61.33±7.40 |
|  | LDH | U/L | 148.40±20.79 | 156.30±26.10 | 132.89±24.62 | 158.56±23.89 |
|  | Ca^2^＋ | mmol/L | 2.44±0.11 | 2.41±0.08 | 2.35±0.11 | 2.36±0.16 |
| Serum | P | mmol/L | 1.04±0.18 | 1.10±0.09 | 1.10±0.17 | 1.21±0.10 |
| Chemistry | K^＋^ | mmol/L | 3.95±0.42 | 3.88±0.15 | 3.84±0.36 | 3.94±0.46 |
| Na＋ | | mmol/L | 140.10±3.11 | 139.60±2.76 | 139.11±1.27 | 141.44±1.51 |
| BUN | | mmol/L | 4.22±1.25 | 3.73±1.10 | 4.21±0.80 | 4.87±1.10 |
| Cr | | umol/L | 82.86±18.34 | 82.68±19.94 | 81.50±8.63 | 71.04±5.42 |
| URIC | | umol/L | 307.70±68.22 | 295.20±101.79 | 354.33±107.10 | 350.56±98.66 |
| Glu | | mmol/L | 4.76±0.31 | 4.69±0.33 | 4.68±0.24 | 4.70±0.18 |
| TC | | mmol/L | 4.20±0.82 | 4.18±0.81 | 3.61±0.47 | 3.65±0.37 |
| TG | | mmol/L | 0.90±0.31 | 1.13±0.57 | 0.82±0.38 | 1.11±0.45 |
| LPS | | U/L | 35.30±8.10 | 38.70±8.25 | 31.78±9.39 | 34.44±10.50 |
| AMY | | U/L | 61.20±12.59 | 64.00±9.51 | 54.89±17.98 | 63.44±12.42 |
| HR | | beat/min | 68.90±8.28 | 66.80±8.00 | 69.11±13.72 | 67.56±17.46 |
| PR | | msec | 147.20±13.56 | 149.50±12.09 | 161.78±48.81 | 160.78±20.42 |
| ECG QRS | | msec | 101.80±12.30 | 102.60±12.62 | 104.56±10.04 | 103.44±10.04 |
| QTc | | msec | 411.60±25.69 | 406.80±24.67 | 410.11±18.41 | 414.78±19.73 |
| RV5+SV1 | | mV | 2.25±0.82 | 2.36±0.87 | 2.43±0.75 | 2.08±0.51 |
| ST segment | |  | Normal | Normal | Normal | Normal |

Abbreviations: **ALB** = albumin; **ALP** = alkaline phosphatase; **ALT** = alanine aminotransferase; **AMY** = amylase; **AST** = aspartate aminotransferase; **BUN** = blood urea nitrogen; **Ca^2+^** = calcium; **Cr** = creatinine; **ECG** = electrocardiogram; **Glu** = glucose; **Hct** = Hematocrit; **HGB** = Hemoglobin; **HR** = Heart Rate; **γ-GT** =gamma-glutamyltransferase; **K^+^** = potassium; **LDH** = lactate dehydrogenase; **LPS** = lipase; **Na^+^** = Sodium; **P** = phosphorus; **PLT** = Platelet; **PR** = PR interval; **QRS** = QRS Interval; **QTc** = Corrected QT interval; **RBC** = red blood cell; **RV5+SV1** = RV5+SV1 amplitude; **SD** = standard deviation; **TBIL** = total bilirubin; **TC** = total cholesterol; **TG** = triglycerides; **TP** = total protein; **URIC** = Uric acid; **WBC** = White blood cell.

## Supplementary Table 15. Laboratory Parameters of the 4.0 μg and 5.0 μg Dosing Groups before and after Treatment of Anfibatide (Mean ± SD)

### 4.0 μg Dosing Group (Group 7) 5.0 μg Dosing Group (Group 8)

|  | Parameter | Unit | Pre-treatment | Post-treatment | Pre-treatment | Post-treatment |
| --- | --- | --- | --- | --- | --- | --- |
|  | RBC | ×10^12^/L | 4.66±0.44 | 4.63±0.43 | 4.75±0.51 | 4.62±0.59 |
|  | WBC | ×10^9^/L | 5.73±1.52 | 5.83±2.06 | 5.69±1.40 | 5.34±0.91 |
| Hematology | PLT | ×10^9^/L | 218.30±47.73 | 229.30±40.82 | 219.90±41.92 | 216.30±41.49 |
|  | HGB | g/L | 143.80±10.00 | 144.60±13.05 | 148.80±12.81 | 145.10±15.86 |
|  | Hct |  | 0.43±0.04 | 0.43±0.04 | 0.44±0.04 | 0.43±0.05 |
|  | TP | g/L | 77.46±3.32 | 80.11±3.39 | 78.29±4.73 | 76.15±3.63 |
|  | ALB | g/L | 47.55±1.24 | 48.57±1.18 | 48.80±2.13 | 48.14±0.98 |
|  | TBIL | umol/L | 17.24±6.20 | 17.07±6.14 | 19.93±4.57 | 18.89±6.95 |
|  | AST | U/L | 19.90±6.92 | 19.00±5.31 | 20.30±2.79 | 20.06±3.95 |
|  | ALT | U/L | 19.90±16.25 | 20.00±13.93 | 12.50±6.47 | 10.90±4.65 |
|  | γ-GT | U/L | 16.40±11.18 | 13.70±9.14 | 13.40±5.93 | 7.00±1.83 |
|  | ALP | U/L | 79.00±25.09 | 83.90±29.53 | 74.00±12.49 | 68.00±12.30 |
|  | LDH | U/L | 146.20±26.19 | 155.30±21.41 | 139.20±15.57 | 142.00±9.49 |
|  | Ca^2^＋ | mmol/L | 2.41±0.12 | 2.58±0.05 | 2.62±0.14 | 2.56±0.03 |
| Serum | P | mmol/L | 1.05±0.19 | 1.16±0.23 | 1.31±0.11 | 1.24±0.08 |
| Chemistry | K^＋^ | mmol/L | 3.69±0.27 | 3.85±0.17 | 4.08±0.39 | 4.00±0.14 |
| Na＋ | | mmol/L | 139.30±3.77 | 141.20±1.81 | 137.60±2.91 | 139.50±1.29 |
| BUN | | mmol/L | 3.99±1.60 | 4.35±1.10 | 4.34±1.01 | 4.67±1.36 |
| Cr | | umol/L | 80.38±13.30 | 82.84±14.31 | 80.55±15.95 | 80.87±15.47 |
| URIC | | umol/L | 301.60±98.66 | 318.60±82.55 | 301.20±78.81 | 244.75±53.92 |
| Glu | | mmol/L | 4.84±0.49 | 5.01±0.35 | 4.97±0.25 | 4.76±0.41 |
| TC | | mmol/L | 3.79±0.63 | 3.88±0.62 | 3.75±0.36 | 3.61±0.24 |
| TG | | mmol/L | 0.90±0.35 | 0.99±0.24 | 0.99±0.35 | 0.72±0.14 |
| LPS | | U/L | 32.40±17.96 | 35.00±10.81 | 44.60±18.54 | 41.25±9.43 |
| AMY | | U/L | 59.60±18.49 | 61.70±17.04 | 63.90±16.66 | 63.25±16.24 |
| HR | | beat/min | 76.00±11.77 | 75.90±11.22 | 66.20±7.55 | 68.30±10.50 |
| ECG PR | | msec | 135.30±12.37 | 134.70±13.15 | 138.70±11.70 | 142.00±12.55 |
| QRS | | msec | 99.70±8.79 | 98.80±9.00 | 105.70±9.43 | 105.20±9.91 |
| QTc | | msec | 418.90±25.56 | 415.20±19.24 | 411.50±21.97 | 412.10±18.91 |
| RV5+SV1 | | mV | 2.71±0.68 | 2.62±0.94 | 2.44±1.00 | 2.39±0.90 |
| ST segment | |  | Normal | Normal | Normal | Normal |

Abbreviations: **ALB** = albumin; **ALP** = alkaline phosphatase; **ALT** = alanine aminotransferase; **AMY** = amylase; **AST** = aspartate aminotransferase; **BUN** = blood urea nitrogen; **Ca^2+^** = calcium; **Cr** = creatinine; **ECG** = electrocardiogram; **Glu** = glucose; **Hct** = Hematocrit; **HGB** = Hemoglobin; **HR** = Heart Rate; **γ-GT** =gamma-glutamyltransferase; **K^+^** = potassium; **LDH** = lactate dehydrogenase; **LPS** = lipase; **Na^+^** = Sodium; **P** = phosphorus; **PLT** = Platelet; **PR** = PR interval; **QRS** = QRS Interval; **QTc** = Corrected QT interval; **RBC** = red blood cell; **RV5+SV1** = RV5+SV1 amplitude; **SD** = standard deviation; **TBIL** = total bilirubin; **TC** = total cholesterol; **TG** = triglycerides; **TP** = total protein; **URIC** = Uric acid; **WBC** = White blood cell.

## Supplementary Table 16. Laboratory Abnormalities of the Single Dose Groups after Treatment of Anfibatide

| *Subject No.* |  | *Parameter* | *Unit* | *Normal Range* | *Pre-treatment* | *Post-Treatment* | *Retested Value* | *Clinical Significance* |
| --- | --- | --- | --- | --- | --- | --- | --- | --- |
| 2A |  | LDH | U/L | 135-225 | 130 | 129 | 147 | No |
| Ca^2+^ mmol/L | | | | 1.9-2.5 | 2.68 | 2.54 | 2.03 | No |
|  |  | AMY | U/L | 0-95 | 90 | 127 | 87 | No |
| 3E |  | LDH | U/L | 135-225 | 128 | 80 | 110 | No |
|  |  | URIC | umol/L | 90-420 | 592 | 592 | 508 | No |
| 3J |  | FOBT |  |  | Negative | (+) | Negative | No |
|  |  | Fecal Analysis |  |  | Yellow and soft, | Yellow and slightly | Yellow and soft, |  |
|  |  |  |  |  | no abnormality | mushy, no abnormality | no abnormality |  |
| 3K |  | WBC | ×10^9^/L | 4-10 | 4.0 | 3.8 | 5.9 | No |
| 4B |  | Urine Protein |  |  | - | 1+ | - |  |
| 5B |  | FOBT |  |  | Negative | Positive (+) | Negative | No |
|  |  | Fecal Analysis |  |  | No abnormality | Small amount of WBC | No abnormality | No |
| 5D |  | WBC | ×10^9^/L | 4-10 | 5.1 | 11.6 | 4.9 | No |
|  |  | HGB | g/L | 120-160 | 157 | 162 | 150 | No |
| 5I |  | TBIL | umol/L | 4.0-26.0 | 20.0 | 29.4 | 26.7 | No |
|  |  | γ-GT | U/L | 0-58 | 7 | 6 | 5 | No |
|  |  |  |  |  |  |  |  |  |
| ＋  Ca2 mmol/L | | | | 1.9-2.5 | 2.39 | 2.52 | 2.43 | No |
| BUN | | | mmol/L | 2.3-7.1 | 2.57 | 2.09 | 2.64 | No |
| 6C **Urinalysis** | | | | | | | | |
| Protein | | |  |  | (-) | (-) | (-) | No |
| WBC | | |  |  | (-) | (-) | (-) | No |
| Occult Blood | | |  |  | (-) | (+-) | (-) | No |
| 7A WBC | | | ×10^9^/L | ~4-10 | 8.2 | 11.0 | 5.2 | No |
| 7E **Urinalysis** | | | | | | | | |
| Protein | | | (+-) | | | 1+ | (-) | No |
| WBC | | | (-) | | | (-) | (-) | No |
| Occult Blood | | | (-) | | | (-) | (-) | No |
| 8A **Urinalysis** | | | | | | | | |
| Protein | | | (-) | | | (-) | (-) | No |
| WBC | | | (-) | | | (-) | (-) | No |
| Occult Blood | | | (-) | | | (+1) | (-) | No |
| 8E **Urinalysis** | | | | | | | | |
| Protein | | | (-) | | | (-) | (-) | No |
| WBC | | | (-) | | | (-) | (-) | No |
| Occult Blood | | | (-) | | | (+1) | (+-) | No |

Abbreviations: **AMY** = amylase; **BUN** = blood urea nitrogen; **Ca^2+^** = calcium; **FOBT** = Fecal Occult Blood Test; **γ-GT** =gamma-glutamyltransferase **HGB** = Hemoglobin; **LDH** = lactate dehydrogenase; **TBIL** = total bilirubin; **URIC** = Uric acid; **WBC** = White blood cell.

**Supplementary Table 17. Laboratory Parameters of the Multiple Dose Groups before and after Treatment of Anfibatide (Mean ± SD)**

### Group 9 Group 10 Group 11

|  | Parameter | Unit | Pre-treatment | Post-treatment | Pre-treatment | Post-treatment | Pre-treatment | Post-treatment |
| --- | --- | --- | --- | --- | --- | --- | --- | --- |
|  | RBC | ×10^12^/L | 5.12±0.13 | 4.88±0.30 | 4.60±0.45 | 4.39±0.42 | 4.69±0.41 | 4.52±0.42 |
|  | WBC | ×10^9^/L | 6.05±0.55 | 6.55±1.02 | 5.28±0.84 | 5.86±1.33 | 5.77±0.96 | 5.09±0.65 |
| Hematology | PLT | ×10^9^/L | 238.33±58.91 | 253.00±45.16 | 232.83±49.12 | 243.08±47.31 | 195.33±40.76 | 213.75±59.31 |
|  | HGB | g/L | 158.50±3.45 | 150.67±7.84 | 141.67±13.92 | 134.33±13.55 | 143.58±13.61 | 135.00±13.70 |
|  | Hct |  | 0.47±0.01 | 0.45±0.03 | 0.42±0.04 | 0.40±0.04 | 0.42±0.04 | 0.41±0.04 |
|  | TP | g/L | 78.18±2.83 | 75.32±2.56 | 77.73±4.32 | 75.82±4.03 | 80.32±5.52 | 77.73±5.60 |
|  | ALB | g/L | 49.18±1.63 | 47.63±1.71 | 49.20±2.39 | 48.89±2.88 | 49.47±2.71 | 47.11±2.26 |
|  | TBIL | umol/L | 15.90±5.88 | 11.83±6.13 | 15.95±6.57 | 13.63±6.45 | 17.65±9.21 | 15.04±6.10 |
|  | AST | U/L | 21.67±4.76 | 22.00±7.04 | 20.27±4.76 | 18.83±5.39 | 21.33±5.42 | 18.17±5.18 |
|  | ALT | U/L | 21.67±16.01 | 25.67±17.12 | 13.45±4.80 | 11.83±3.97 | 15.92±8.72 | 15.33±8.39 |
|  | γ-GT | U/L | 17.83±7.14 | 22.50±8.96 | 12.55±4.23 | 11.92±3.80 | 17.33±14.57 | 14.92±5.40 |
|  | ALP | U/L | 90.67±34.83 | 85.17±30.66 | 68.75±23.40 | 65.75±20.28 | 74.17±25.28 | 69.08±24.64 |
|  | LDH | U/L | 162.00±11.64 | 161.50±23.25 | 150.42±32.73 | 150.08±28.52 | 151.33±15.13 | 145.25±11.27 |
|  | Ca^2^＋ | mmol/L | 2.68±0.16 | 2.57±0.05 | 2.54±0.13 | 2.60±0.07 | 2.45±0.15 | 2.44±0.07 |
| Serum | P | mmol/L | 1.19±0.12 | 1.22±0.21 | 1.20±0.16 | 1.19±0.13 | 1.25±0.15 | 1.19±0.08 |
| Chemistry | K^＋^ | mmol/L | 4.28±0.19 | 3.98±0.29 | 4.24±0.35 | 4.03±0.31 | 4.12±0.26 | 4.11±0.24 |
| Na＋ | | mmol/L | 142.33±1.03 | 142.67±2.58 | 143.33±2.19 | 142.08±2.19 | 141.42±3.40 | 144.92±1.83 |
| BUN | | mmol/L | 4.84±1.56 | 4.74±1.18 | 4.68±0.80 | 5.18±0.95 | 4.88±1.24 | 4.98±1.20 |
| Cr | | umol/L | 84.18±3.95 | 79.32±7.00 | 71.89±16.07 | 68.88±12.69 | 67.91±10.26 | 70.93±10.15 |
| URIC | | umol/L | 352.67±27.35 | 351.33±45.07 | 302.27±56.31 | 313.08±70.70 | 289.25±68.28 | 294.33±101.43 |
| Glu | | mmol/L | 4.89±0.60 | 5.03±0.29 | 4.84±0.30 | 4.99±0.56 | 4.98±0.27 | 4.98±0.39 |
| TC | | mmol/L | 4.13±0.94 | 3.87±0.87 | 3.78±0.55 | 3.72±0.60 | 4.09±0.37 | 4.07±0.58 |
| TG | | mmol/L | 1.01±0.46 | 1.14±0.60 | 0.72±0.23 | 0.85±0.28 | 0.89±0.26 | 1.05±0.81 |
| LPS | | U/L | 45.50±46.49 | 46.33±12.16 | 50.33±23.52 | 51.00±18.67 | 34.83±3.49 | 38.83±14.98 |
| AMY | | U/L | 63.50±17.44 | 64.33±22.12 | 62.08±18.07 | 60.42±14.87 | 64.25±16.84 | 60.17±18.48 |
| HR | | beat/min | 70.50±9.97 | 70.67±11.09 | 70.50±11.75 | 69.00±7.77 | 78.58±15.62 | 66.75±8.97 |
| ECG PR | | msec | 146.83±13.57 | 144.67±11.11 | 151.33±17.97 | 150.67±18.86 | 144.42±11.77 | 154.00±11.62 |
| QRS | | msec | 103.00±8.12 | 105.00±4.05 | 101.33±7.13 | 103.25±8.16 | 100.92±9.30 | 98.42±8.70 |
| QTc | | msec | 403.33±10.76 | 400.17±20.75 | 413.50±21.38 | 412.17±14.50 | 432.75±19.78 | 415.42±15.22 |
| RV5+SV1 | | mV | 2.87±0.71 | 2.73±0.80 | 2.04±0.86 | 1.83±0.68 | 2.20±0.55 | 2.03±0.44 |
| ST segment | |  | Normal | Normal | Normal | Normal | Normal | Normal |

Abbreviations: **ALB** = albumin; **ALP** = alkaline phosphatase; **ALT** = alanine aminotransferase; **AMY** = amylase; **AST** = aspartate aminotransferase; **BUN** = blood urea nitrogen; **Ca^2+^** = calcium; **Cr** = creatinine; **ECG** = electrocardiogram; **Glu** = glucose; **Hct** = Hematocrit; **HGB** = Hemoglobin; **HR** = Heart Rate; **γ-GT** =gamma-glutamyltransferase; **K^+^** = potassium; **LDH** = lactate dehydrogenase; **LPS** = lipase; **Na^+^** = Sodium; **P** = phosphorus; **PLT** = Platelet; **PR** = PR interval; **QRS** = QRS Interval; **QTc** = Corrected QT interval; **RBC** = red blood cell; **RV5+SV1** = RV5+SV1 amplitude; **SD** = standard deviation; **TBIL** = total bilirubin; **TC** = total cholesterol; **TG** = triglycerides; **TP** = total protein; **URIC** = Uric acid; **WBC** = White blood cell.

## Supplementary Table 18. Laboratory Abnormalities of the Multiple Dose Groups after Treatment of Anfibatide

| Subject No. |  | Parameter | Unit | Normal Range | Pre-treatment | Post-Treatment | Retested Value | Clinical Significance |
| --- | --- | --- | --- | --- | --- | --- | --- | --- |
| 9A |  | HGB | g/L | 120-160 | 163 | 156 | 147 | No |
|  |  | Ca^2^＋ | mmol/L | 1.9-2.5 | 2.36 | 2.64 | 2.03 | No |
| 9B |  | Hct |  | 0.380-0.508 | 0.477 | 5.09 | 87 | No |
|  |  |  |  |  | **Urinalysis** |  |  |  |
| 10K |  | Protein |  |  | (-) | (-) |  | No |
|  |  | WBC |  |  | (-) | (-) |  | No |
|  |  | Occult Blood |  |  | (-) | (++) | (-) | No |
|  |  |  |  |  | **Urinalysis** |  |  |  |
| 10L |  | Protein |  |  | (-) | (-) |  | No |
|  |  | WBC |  |  | (-) | A few | (-) | No |
|  |  | Occult Blood |  |  | (-) | (+) | (-) | No |

Abbreviations: **Ca^2+^** = calcium; **Hct** = Hematocrit; **HGB** = Hemoglobin; **WBC** = White blood cell.

# Supplementary Figures

|  | **Index** | **Page No.** |
| --- | --- | --- |
| **Supplementary Figure 1A.** | **Mean AUC(0-t) and AUC(0-∞) Versus Dose of Anfibatide after Administration of 1, 1.5, 2, 3, 4 and 5 µg/60kg Doses, Respectively** | **20** |
| **Supplementary Figure 1B.** | **Positive Correlation between AUC and Dose** | **20** |
| **Supplementary Figure 2.** | **Anfibatide Specifically Inhibited Human Platelet Aggregation Induced by Ristocetin (1.5 mg/mL) but not ADP (10 μM), Collagen (4 µg/mL) or PAR1AP (10 μM)** | **21** |
| **Supplementary Figure 3.** | **Anfibatide Inhibited Human Platelet Aggregation Induced by Botrocetin (20μg/mL) dose dependently** | **22** |

| 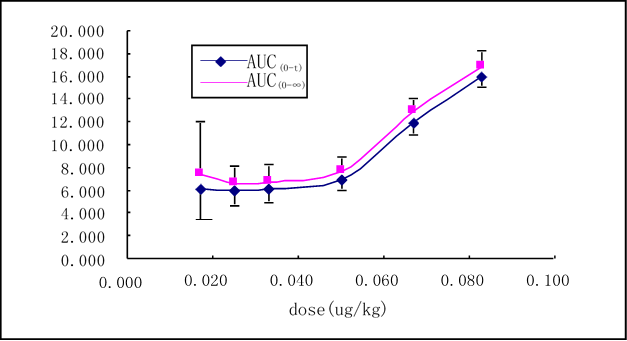  AUC(ug.h/ml) | 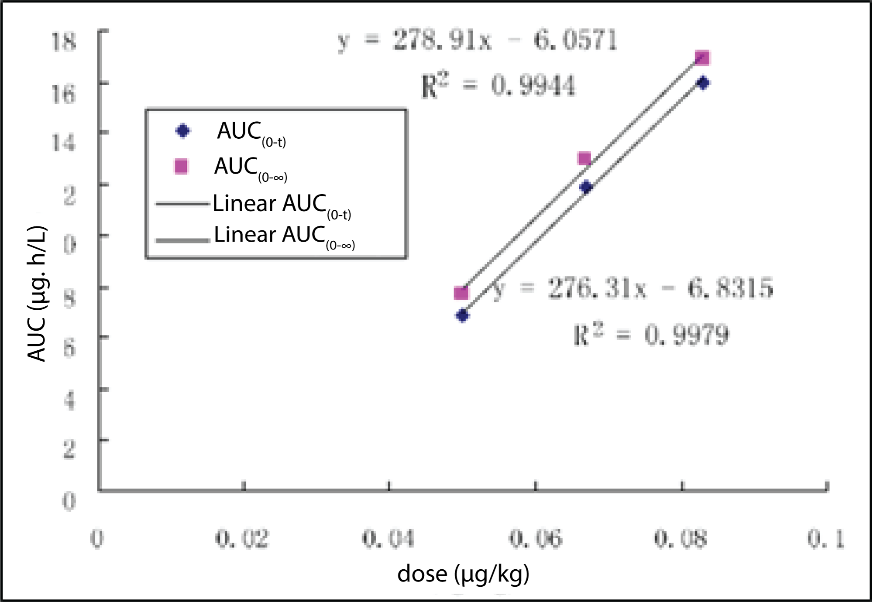 |
| --- | --- |
| Supplementary Figure 1A. Mean AUC_(0-t)_ and AUC_(0-∞)_ versus Dose of Anfibatide after Administration of 1, 1.5, 2, 3, 4 and 5 µg/60kg Doses, Respectively. AUC_(0-t)_, area under the plasma concentration-time curve from time 0 to time t; AUC_(0-∞)_, area under the plasma concentration-time curve from time 0 to infinity. | **Supplementary Figure 1B. Positive Correlation between AUC and Dose.** AUC, area under the plasma concentration-time curve. |


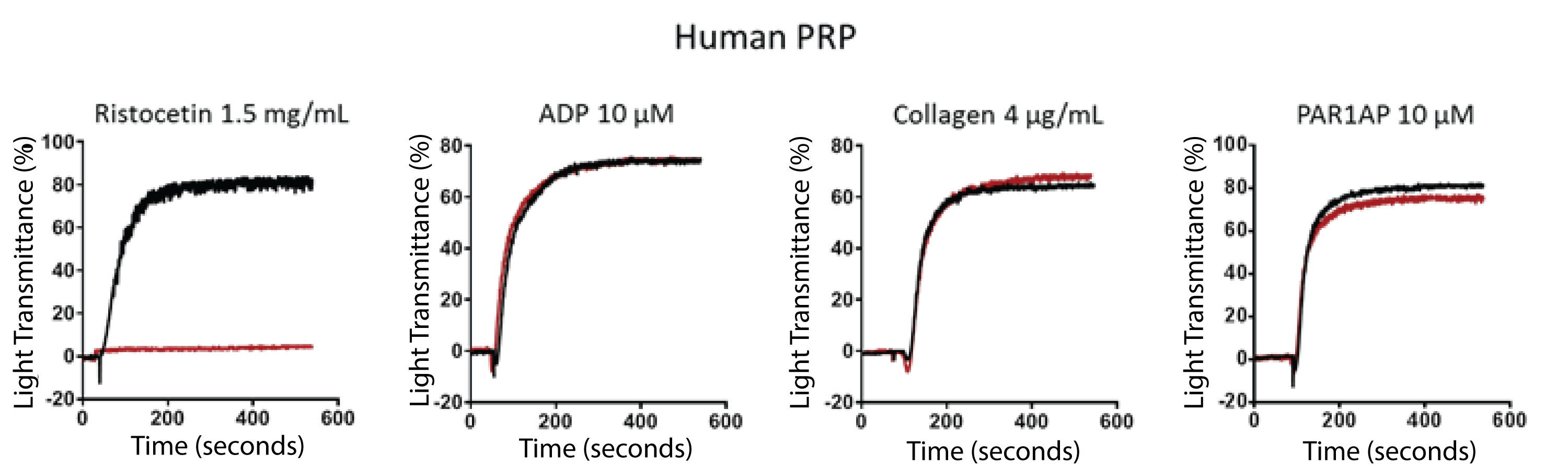


**Supplementary Figure 2.** **Anfibatide Specifically Inhibited Human Platelet Aggregation Induced by Ristocetin (1.5 mg/mL) but not ADP (10 μM), Collagen (4 µg/mL) or PAR1AP (10 μM).** PRP, platelet-rich plasma; ADP, adenosine diphosphate; PAR1AP, PAR1 receptor activating peptide. Red curve: anfibatide treated PRP; Black curve: control PRP

Human PRP

**Time (seconds)**

**Light transmittance (%)**

**Supplementary Figure 3.** **Anfibatide Inhibited Human Platelet Agglutination/Aggregation Induced by Botrocetin (20μg/mL) dose dependently.** PRP, platelet-rich plasma
